# Brazilian forensic nursing from the perspective of its experts

**DOI:** 10.1590/1980-220X-REEUSP-2024-0402en

**Published:** 2025-05-02

**Authors:** Davydson Gouveia Santos, Vanessa Martinhago Borges Fernandes, Morgana Oliveira Citolin, Andreza Goulart Hilleshein, Maria de Fátima Saturnino, Mara Ambrosina de Oliveira Vargas

**Affiliations:** 1 Universidade Federal de Santa Catarina, Programa de Pós-Graduação em Enfermagem, Florianópolis, SC, Brazil.

**Keywords:** Forensic Nursing, Nursing Care, Science and Development, Nurse’s Role, Nursing, Practical

## Abstract

**Objective:**

To understand Brazilian forensic nursing from experts’ perspective.

**Method:**

A qualitative study, carried out with nurses specialized in forensic nursing. Data collection was done through an electronic questionnaire applied between February and May 2023, composed of questions about socio-professional and performance characteristics. The data were organized by lexicographic analysis by IRAMUTEQ, analyzed from the perspective of Bardin’s content analysis.

**Results:**

Fifty-five experts who worked in the five Brazilian regions participated, with the largest participation from the Northeast and Southeast regions, both with 41.8% of professionals. The majority reported being women, white, mostly married or in a stable union. From the association of classes presented in the dendrogram generated by IRAMUTEQ, five categories related to the Descending Hierarchical Classification lexical terms were generated: 1) Creation of forensic nursing and its political development in national territory; 2) Establishment of environments for forensic nursing practice according to national/cultural reality; 3) Already established competencies of Brazilian forensic nursing; 4) Paths to success in Brazilian forensic nursing; and 5) Perspectives for Brazilian forensic nursing.

**Conclusion:**

The study allowed contextualizing the current scenario of Brazilian forensic nursing. The results revealed violence as the focus of the actions developed by the specialty. Finally, it was possible to relate forensic nursing experts’ knowledge and experiences, enabling the understanding of the path already taken and what still needs to be followed, such as the development of public policies that include forensic nursing practice.

## INTRODUCTION

Forensic sciences are a set of technical and scientific bases applied to investigation of crimes and situations of violence with an approach focused on the legal aspect. The term “forensic science” began to be inserted into health sciences intentionally to train professionals in various areas, especially nursing^(1)^.

In Brazil, Resolution 389/2011 of the Federal Nursing Council (In Portuguese, *Conselho Federal de Enfermagem* - COFEN) recognizes forensic nursing as a specialty of the professional category among the 65 existing specialties^(2)^. COFEN Resolution 556/2017 regulated Brazilian forensic nurses’ practice, their areas of activity and the technical competencies of the specialty^(3)^.

According to this resolution, forensic nurses can work in a wide range of situations, including trauma, violence, sexual abuse and drug use as well as psychiatric pathologies. Their work includes providing assistance to various groups, such as aggressors, vulnerable populations and the prison system. It includes activities such as expert assessments and consultancies, as well as working in mass disasters and different forms of violence, often outside of hospital settings. To perform this role, forensic nurses must have a specialization degree recognized by the Ministry of Education or by institutions linked to the COFEN/Regional Councils System^(3)^.

Forensic nurses’ fundamental competencies include preserving evidence and the chain of custody, obtaining data through photographs and documentation, and preparing reports and opinions for the courts. Moreover, nurses are responsible for providing consultancy in cases involving the forensic area, including health issues, bodily injuries, fraud and other forms of abuse^(4)^.

Forensic nurses’ work is quite diverse, crucial in caring for victims of violence, as nursing professionals normally provide the first support to patients in a hospital environment^(4)^. Furthermore, violence has progressively increased over the years, reflecting a complex interaction of social, economic, political and cultural factors^(5)^. As a systemic phenomenon, violence directly influences the health, security and social security systems, with a negative impact on the population’s quality of life.

The development of forensic nursing in Brazil can significantly contribute to improving victims’ quality of life and helping to prevent episodes of violence^(6)^. Nurses play an essential role in caring for victims of violence, being indispensable in anamnesis, in carrying out physical examination and in nursing diagnosis. These stages are decisive in the entry into the health system^(7)^. Regardless of the length of experience in the health field, nurses recognize the importance of forensic training in the clinical context to ensure compliance with ethical and legal principles and to take responsibility for the recognition, collection and preservation of evidence in the context of assisting users with complex physical, psychological and psychosocial needs^(8, 9)^.

Given the crucial role of nurses in patient care and support, they become a central figure in the process of assisting victims of violence within the context of care. Forensic nursing is different because it links the health system with the judicial system, involving actions such as searches, investigations and care. In addition to clinical practice, this area of nursing plays an important role in collecting evidence by actively engaging in the prevention, identification and combat of violence, contributing to the development of responses to different situations of aggression^(10)^.

By applying forensic nursing knowledge, nurses can identify cases of violence and promote more humane care, since their actions can help break the cycle of violence. To do so, they need to be adapted to individual needs, based on scientific evidence and developed in accordance with current laws, public health policies and basic nursing resources, with the aim of reducing the harm caused to victims of violence^(11)^.

Given the above, and because it is an emerging area that has not yet been incorporated into Brazilian health institutions, this article aimed to understand Brazilian forensic nursing from experts’ perspective. To this end, historical aspects, the current reality and the prospects for advancement of the specialty were considered.

## METHOD

### Study Design

This is a descriptive study, with an exploratory approach and of a qualitative nature. The presentation followed the COnsolidated criteria for REporting Qualitative research (COREQ) recommendations^(12)^.

### Local

Convenience sampling was used, and data were collected through the distribution of an online questionnaire on virtual platforms such as email, Instagram^®^, Facebook^®^, and WhatsApp^®^ groups. The questionnaire was made available on Google Forms^®^, and this approach allowed us to reach all regions of Brazil.

### Population and Selection Criteria

The study population was composed of forensic nurses with practical experience in care or teaching in national territory, which included people from different regions of the country and allowed the understanding of regional variations.

The inclusion criteria for participating in the study were to be forensic nurses with a *lato sensu* specialization in forensic nursing or expertise in the area, with practical experience in care or teaching in national territory. The exclusion criterion included professionals who had an interest in the area, but did not have theoretical and practical qualifications related to forensic nursing.

### Sample Definition

The snowball technique, a non-probabilistic sampling, was used to recruit participants. Referral chains were used to locate potential participants. Initially, the first individuals were invited through referrals from the first author of this study and, subsequently, through suggestions from the participants themselves. Data collection was stopped when referrals began to be repeated, with no additional responses to the online questionnaire. In total, 63 responses were recorded, and after data refinement, 55 forensic nurses met the inclusion criteria and completed the questionnaire.

### Data Collection

Data were collected between February and May 2023, using a structured questionnaire developed by the authors, which was divided into four parts. The first part contained the research title, the invitation to participate, the inclusion criteria, the Informed Consent Form, and the options “I have read the terms and agree to participate in the research” and “I have read the terms and do not agree to participate in the research”. When selecting the agreement option, participants had access to the questionnaire, while, otherwise, the invitation was closed and Google Forms^®^ redirected them to a thank you page. The second part addressed questions about participant characteristics. The third part applied the Free Word Association Technique (FWAT), which asked participants to mention the five words that came to mind when thinking about the role of forensic nurses in Brazil. FWAT is a subjective technique used to explore latent content not filtered by censorship. The fourth part addressed the application of forensic nursing in Brazil.

### Data Analysis and Treatment

The socio-professional profile data were recorded in a Microsoft Excel^®^ spreadsheet, with double checking, and processed using the Statistical Package for the Social Sciences (SPSS) version 29. The analysis followed descriptive statistics, with measures of central tendency (mean, median, maximum and minimum) and dispersion (standard deviation). The data obtained through FWAT and on the development of forensic nursing in Brazil were analyzed lexicographically, using the *Interface de R pour les Analyses Multidimensionnelles de Textes et de Questionnaires* (IRAMUTEQ).

IRAMUTEQ^(13)^ is a tool that organizes texts based on similarity between words, allowing the creation of classes and categories that group the most significant terms. These results are presented in the form of a word cloud and dendrogram, using the Descending Hierarchical Classification (DHC). To interpret DHC data, active forms (adjectives, adverbs, nouns, verbs) of each segment class were analyzed. Texts with a value ≥ 3.26 and p-value ≤ 0.03433 in the chi-square test (chi**2**) indicate statistical significance in the association of words within their class.

After processing, the core meaning of segments was analyzed, highlighting the most significant words. This made it possible to categorize lexical content in responses, organized by thematic axes through content analysis. This type of analysis involves reading the evidence produced, coding the units of meaning, interpreting and formulating inferences, in addition to analyzing the thematic categories generated by IRAMUTEQ, establishing connections with the research conceptual frameworks.

The data from the fourth stage were analyzed using the content analysis technique proposed by Bardin^(14)^, with the support of Qualitative Data Analysis (QDA) Miner^(15)^. Content analysis comprises the “set of communication analysis techniques” and involves the stages of pre-analysis; material exploration; data processing and interpretation.

In pre-analysis, the data were transferred to QDA Miner, and skim readings were performed to understand the text and select indicators for the next phase. In material exploration, the coding and condensation of recording units were performed to construct the empirical categories. Then, the data were processed and interpreted according to the reading on the topic.

### Ethical Aspects

The study was approved by the *Universidade Federal de Santa Catarina* Research Ethics Committee, under Opinion 5.808.287. All participants signed the Informed Consent Form online, by selecting the option “I have read the terms and agree to participate in the research”. All ethical aspects of research involving human subjects were met. To ensure anonymity, participants were identified by the abbreviation “FN” (forensic nurse), followed by a cardinal number in ascending order.

## RESULTS

A total of 55 (100%) nurses participated in the study, 46 (83.6%) female and nine (16.4%) male, with a mean age of 41 years (SD = 10.5; Min = 23; Max = 65). Regarding race, 29 (52.7%) declared themselves white, 18 (32.7%) were brown, seven (12.7%) were black, and one (1.8%) was yellow. The majority of them were married or in a stable union (n = 26/47.3%). Moreover, 22 (40%) were single, six (10.9%) were divorced, and one (1.8%) was widowed. The predominant religion was Catholicism, cited by 30 (54.5%) participants; six (10.9%) declared themselves Protestant or evangelical; five (9.1%) declared themselves agnostic; four (7.3%) declared themselves to be spiritualists; six (10.92%) professed other Christian or African-origin beliefs (Christian, Jehovah’s Witness, *Candomblé, Umbanda*); and four (7.2%) declared themselves to be atheists or to have no religious beliefs.

Concerning the time of graduation, 32 (58.2%) reported between six and 20 years of professional experience, 18 (32.7%) between 21 and 40 years, and only five (9.1%) up to five years. In addition to this, 14 (25.5%) held a doctoral degree, ten (18.2%), a master’s degree, and 31 (56.3%), a specialization degree. Table 1 presents the training profile of these professionals.

**Table 1:** Profile of nursing training for improvement in forensic nurses Florianópolis, SC, Brazil, 2024.

Variables		N	%
**Nursing training time**	Up to five years of training	5	9.1
	Six to ten years of training	12	21.8
	11 to 20 years of training	20	36.4
	21 to 30 years of training	11	20
	Over 30 years of training	7	12.7
	Total	55	100
**Highest degree**	Doctorate	14	25.5
	Master	10	18.2
	Expert	31	56.3
	Total	55	100
**Year of training in forensic nursing**	2015	2	3.6
	2016	1	1.8
	2018	9	16.4
	2019	16	29.1
	2020	2	3.6
	2021	8	14.5
	2022	9	16.4
	2023	3	5.5
	2024	5	9.1
	Total	55	100
**How did you acquire the title of forensic nursing expert?**	In-person specialization	19	34.5
	Distance Learning Specialization (EAD)	11	20
	Proof of qualifications from professional associations representing the area	20	36.4
	Studying + free courses	5	9.1
	Total	55	100
**Institution that conferred the title of forensic nurses**	ABEFORENSE	8	14.5
	SOBEF	11	20
	IDE – Christ the Redeemer University	8	14.5
	Keynes Institute	6	10.9
	UNIFAJ	5	9.1
	Unyleya	4	7.3
	Others	13	23.7
	Total	55	100
**Professional performance by national region**	Central-West Region	3	5.5
	Northeast Region	23	41.8
	North Region	1	1.8
	Southeast Region	23	41.8
	South Region	5	9.1
	Total	55	100

Source: survey data. 2023.

Among the study participants, 28 (50.9%) were registered as forensic nursing experts with the COREN to which they were linked. As for their affiliation with representative entities in the area, 20 (36.4%) were associated with SOBEF, 12 (21.8%), with ABEFORENSE, and 23 (41.8%) were not linked to representative bodies in the area.

**Figure 1:**
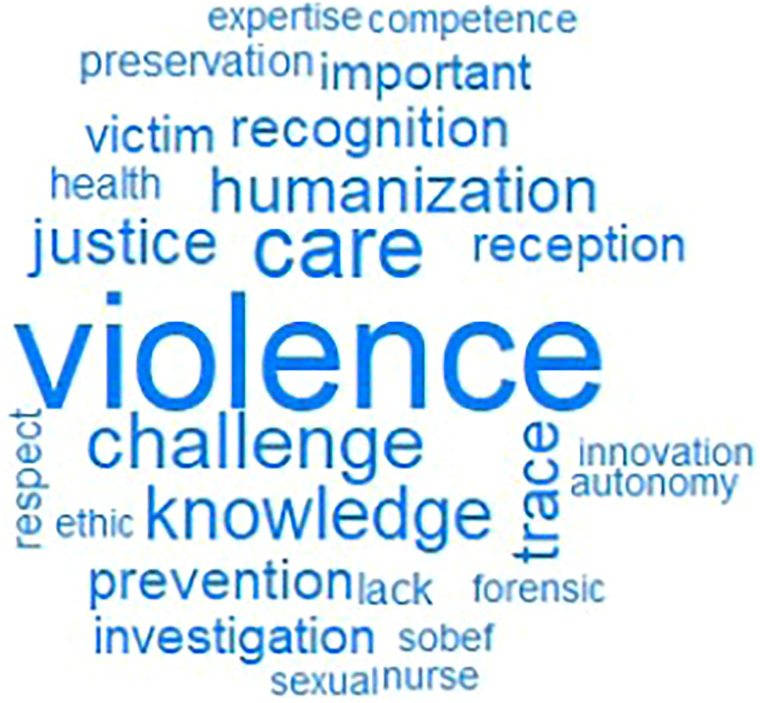
Word cloud generated by the Free Word Association Technique about the role of forensic nursing in Brazil. Brazil, 2023.

Source: IRAMUTEQ software, 2023.

FWAT was performed by citing five words when thinking about “forensic nurses’ work in Brazil”. The cognates evoked were recorded in the order in which they were mentioned. A total of 275 words/expressions were evoked by 55 nurses, and 29 distinct word-expressions were identified as being the most frequently used. The *corpus* of evocations was analyzed in IRAMUTEQ, using the word cloud technique, which is one of the data processing methods applied in this study. This approach allows the graphical presentation of the most frequent words, with the size of each term being proportional to the number of times it was mentioned. Among the most frequently used keywords during data collection, the term “violence” stood out, mentioned by 21 of participants. Terms mentioned less than twice were excluded from the graph. Thus, Figure 1 was generated.

Word cloud was used as a starting point for data analysis. The words with less prominence indicate different aspects that influence the forensic nursing scenario, establishing direct connections with the most prominent terms. Among the most recurrent terms, “violence” (21), “care” (11), “challenge” (eight) stand out, in addition to “justice”, “humanization” and “traces”, which were mentioned seven times each. DHC allowed the segmentation of the text *corpus* into classes of text segments (TSs) and their respective words, highlighting the central ideas present in participants’ responses. The hierarchical analysis resulted in 151 TSs, of which 132 were classified, obtaining a utilization of 87.42% and forming five classes. In other words, five groupings of TSs were created with similar and correlated words among themselves, but distinct from the others.

**Figure 2:**
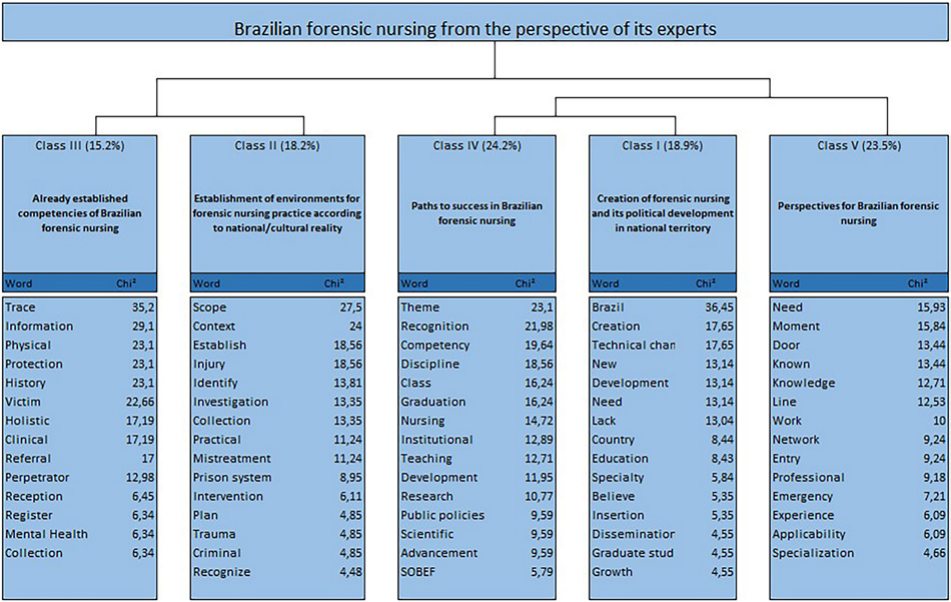
Dendrogram of the Descending Hierarchical Classification with significant words about the development of Brazilian forensic nursing. Brazil, 2023.

Source: prepared by the authors, based on data generated by the IRAMUTEQ software, 2023.

In the dendrogram, the text *corpus* was divided into two groups: the first, composed of classes II and III, accounting for, respectively, 18.2% and 15.2% of TSs; the second group, formed by a subdivision of class V, corresponding to 23.5% of TSs, originating classes I and IV, which account for 18.9% and 24.2% of TSs, respectively (Figure 2).

After processing the *corpus* using the software, it was found that classes I, IV and V are associated with each other and are opposed, in lexical terms, to classes II and III. The TSs of each cluster were retrieved and interpreted in detail, based on the principles of thematic analysis, aiming to understand their core meanings and thus categorize the classes. Based on the results presented in the DHC dendrogram, the categorized classes were described and exemplified with excerpts taken from the questionnaire.

Class I (Creation of forensic nursing and its political development in national territory), related to the milestones of creation and development of the specialty in Brazil, gathered 25 TSs (18.9%) of the total *corpus* analyzed, consisting of words and radicals in the range between x^2^ = 4.55 (growth) and x^2^ = 36.45 (Brazil), such as “creation” (x^2^ = 17.65), “elaboration” (x^2^ = 13.14) and “specialty” (x^2^ = 5.84).

Forensic nurses mentioned the emergence and development of the specialty, and related elements of training with professional practice to strengthen the area: [...] *Resolution 556/2017 of COFEN* (FN32); *Preparation of protocols and judicial requirements* (FN03); [...] *creation of projects to promote the creation of positions for forensic nurses in Minas Gerais* (FN20); [...] *since with the increase in graduate courses the number of experts becomes greater and makes the area stronger* (FN50); [...] *the nursing professional is the first professional to approach patients in the hospital unit; therefore, they have a lot to contribute to the collection of evidence and information to be delivered to the justice system* (FN16); *I entered the graduate course and I have been enchanted by the world of forensic* [nursing] *and the power of this specialty; I try to put it in everything I can* (FN17); [...] *I am sure that there will be a before and after in Brazilian society after the creation of the position and forensic nurses’ work in the area* (FN42).

Class II (Establishment of environments for forensic nursing practice according to national/cultural reality) concerns possible services provided by the specialty, sometimes characterized by the fragility of those being treated. It covered 24 TSs (18.2%) of the total *corpus* analyzed, and was made up of words and radicals in the range between x^2^ = 4.48 (recognize) and x^2^ = 27.5 (scope), such as “context” (x^2^ = 24), “injury” (x^2^ = 18.56), “identify” (x^2^ = 13.81) and “intervention” (x^2^ = 6.11).

The analysis revealed the need for nursing professionals to apply forensic knowledge in existing environments, who sometimes do not apply forensic care: [...] *provide consultancy support in cases of litigation related to the forensic area in the scope of health care, civil liability for bodily injuries and other abuses;* [...] *perform prison forensic nursing activities with offenders, judicially penalized, focusing on an interdisciplinary approach in decision-making with the judicial and public security system, within the scope of criminal, civil, military and labor law, with the objective of resocialization;* [...] *collect, gather and preserve evidence, in the victim and the perpetrator, in the different contexts of forensic nursing practice, in pre-hospital, hospital, community settings, in Basic Prison Health Units* (BPHU) *or other professional contexts, in compliance with applicable legal limits;* [...] *participate in measures to preserve bodies in the context of mass disasters, riots, catastrophes and humanitarian missions;* [...] *provide support to victims and potential victims in situations of sexual violence, provide forensic nursing care and recognize the importance of the victim’s participation in the collection, gathering and preservation of evidence* [...] (FN18); *There is a lack of knowledge among people about forensic nursing. There is a need to create formal jobs to work as a forensic nurse, as well as develop and disseminate forensic nursing as a science and work as a health professional in the various environments where its work is necessary* (FN29); *and Reach professional environments* (FN43).

Class III (Already established competencies of Brazilian forensic nursing) presents the elements that are already included in current competencies, but it is necessary to go further, expanding them according to the possibilities that the area has. It gathered 20 TSs (15.2%) of the total *corpus* analyzed, and was constituted by words and radicals in the range between x^2^ = 6.34 (collection) and x^2^ = 35.2 (traces), such as “information” (x^2^ = 29.1), “protection” (x^2^ = 23.1) and “perpetrator” (x^2^ = 13.98).

Competencies can be expanded as the area becomes more widespread and as qualifications of professionals working in services that provide assistance to victims and perpetrators increase: *When I began my studies for the specialization* (forensic nursing), *I listed the competencies of the forensic nurse and added some: applying nursing processes in the care of the prison population in the prevention of mistreatment, sexual violence and other forms of violence;* [...] *providing consultancy support in cases of litigation related to the forensic area in the scope of health care, civil liability for bodily injuries and other abuses; Issuing informative opinions, as a forensic consultant, on the provision of health care and the relevant results;* [...] *validate the collection of traces made by forensic nurses* (FN18); [...] *education of patients and family members to recognize expressions of violence and activate the support network; activate the interprofessional team for the protection and advocacy of the patients assessed; education for nonviolent communication; formation of support groups and reflective groups in work based on gender, sexuality and masculinity to confront violence* (FN5); *Nursing reception and assistance to victims/perpetrators/families; referrals to the protection network; collection and preservation of evidence and exams; assessment and recording of expert reports; interventions and training of professionals and the general population to prevent violence* (FN34).

Class IV (Paths to success in Brazilian forensic nursing) brings together actions that, when implemented, can favor the consolidation of the specialty in Brazil. It was composed of 32 TSs (24.2%) of the total *corpus* analyzed, consisting of words and radicals in the range between x^2^ = 5.79 (SOBEF) and x^2^ = 23.1 (theme), such as “recognition” (x^2^ = 21.98), “institutional” (x^2^ = 12.89), “public policies” (x^2^ = 9.59) and “scientific” (x^2^ = 9.59).

Professionals who carry out practical activities related to the forensic area highlighted its importance, in addition to the need to strengthen public policies and advance teaching at different academic levels: *Recognition in the area of research/academics* (FN2); *Recognition of the importance of applying forensic expertise in public health contexts* (FN4); *Professional recognition through case elucidation* (FN18); *Recognition from other colleagues in forensic sciences* (FN30); *Social and institutional recognition; public policies; qualified educational institutions for training and qualification; validation of protocols following due rigor; coordination of services and professionals* (FN34); *Being recognized in the organizational charts of assistance services and having a greater role in the area of violence* (FN54).

Class V (Perspectives for Brazilian forensic nursing) reflects the positive desire for evolution for the specialty as well as the difficulties of professionals in its consolidation. Composed of 31 TSs (23.5%) of the total *corpus* analyzed, it was constituted by words and radicals in the range between x^2^ = 4.66 (specialization) and x^2^ = 15.93 (need), such as “moment” (x^2^ = 15.84), “knowledge” (x^2^ = 12.71), “work” (x^2^ = 10) and “professional” (x^2^ = 9.18).

The desire for the specialty to grow was expressed by the difficulties that the area has faced, but also by its potential: *Forensic nursing has enormous potential for growth and action in Brazil due to the need for support and assistance to victims of violence that is so prevalent in our country* (FN29); *Honestly, they are small. Due to what I said above, unfortunately, Brazil is a country frozen in its principles and has to fight hard to achieve anything, but I would like to believe that one day we will be like the US and have a truly active forensic nursing* (FN17); *Get in touch with professionals with knowledge in the area and expand the profession in our state of Santa Catarina. Currently, forensic nursing is concentrated with most of the most respected professionals in the northeast of the country, and we need to insert forensic nurses in the entire network and health institutions* (FN37); *Very low, as I do not see public policies or authorities fighting for our profession* (FN24); *and Recognition of the specialty as a way to combat violence and insertion of public policies contemplating forensic nursing* (FN47).

## DISCUSSION

This study included the process of insertion of forensic nursing in the Brazilian scenario, the environments in which specialty practices can and should be exercised, as well as the possibilities of expanding established competencies, the paths that the area needs to follow for national consolidation and the perspectives of professionals that are related to overcoming existing challenges for the application of knowledge.

Forensic nursing care involves the application of practical competencies and critical thinking about the nursing process to positively impact the health of patients affected by trauma or violence, through the identification and recording of forensic evidence that must be presented to the criminal justice system regarding the care provided to individuals^(16)^.

Research into forensic nursing practices is being conducted worldwide^(16, 17, 18)^. In Brazil, studies have been developed relating the possibilities of implementing forensic practices during care already provided by generalist nurses and experts in other areas, such as emergency, obstetrics, sexual violence, prison system^(9, 19, 20)^, among others. There is a need to include experts in specialized services to care for people in vulnerable or violent situations as well as the perpetrators of these situations.

This study converges with others on the importance of promoting awareness about forensic nursing, recently regulated in Brazil, as well as its different areas of activity^(9, 21)^. Although specific training in this sector is still limited, nursing professionals provide care in accordance with established guidelines, often guided by institutional protocols. This highlights the strategy used in caring for victims of violence, and nurses perform the role of welcoming and guiding patients to other specialties^(19)^.

Violence is at the heart of forensic nurses’ work, whether in prevention, control or treatment of victims or perpetrators of violence. Classically, violence is designated by the World Health Organization as a serious public health problem that is preventable. It is defined as “the intentional use of physical force or power, threatened or actual, against oneself, another person, or against a group or community, that either results in or has a high likelihood of resulting in injury, death, psychological harm, maldevelopment or deprivation”^(21)^. Providing the care to be provided becomes a challenge for forensic nursing professionals.

Forensic nursing is a new specialty that combines scientific knowledge and stands out in criminal investigations. However, scientific knowledge is required to successfully fulfill this responsibility. It is important that professionals have adequate knowledge and sensitivity to identify violence and, through their intervention, help promote justice^(22)^. Nurses on the front line of care provide first aid to victims of violence, regardless of how long they have been working in the sector or whether they consider themselves capable or prepared for this type of care. Furthermore, there is a shortage of human resources and a lack of recognition of the role of forensic nursing among the challenges of nursing practice^(23)^. The perception of nurses who collect, identify and preserve evidence when caring for patients in situations of violence facilitates the legal procedures of cases that are brought to court.

Forensic nursing is the result of the combination of nursing science with the health and justice systems. Its history began in the 1970s in the United States, when a group of nurses who defended women’s rights fought for more comprehensive care for rape victims, which included medical evidence collection. Nurses performed physical examinations and collected forensic evidence while treating victims of sexual violence, but they were not recognized as experts and were not allowed to give testimony to judicial authorities in court^(23)^.

With the introduction of the Sexual Assault Medical Forensic Exam program in the United States in the 1980s, nurses were recognized as examiners, establishing a new field of expertise for the profession^(24)^. Given the growing need for care for victims of violence, it was essential for nurses to develop new competencies and obtain specific certifications to work in the forensic field. Thus, they began to act as independent examiners in teams made up of various experts, including police officers, prosecutors and public defenders. This pioneering model originated in California, in the United States, through the Sexual Assault Nurse Examiner and Sexual Assault Response Teams programs^(25)^.

The development and advancement of the forensic nursing specialty in Brazil represent important milestones. With greater progress observed in nations such as Portugal, the United States and Japan, the specialty is in its early stages in Latin America and is still in its infancy in the country. However, it is already officially recognized as a specialization for nurses by COFEN, as established by Resolution 389/2011, later replaced by COFEN Resolution 581/2018^(2)^. Brazilian forensic nursing is regulated by Resolution 556/2017, which defines the guidelines for practice. This resolution establishes the areas of activity as well as the general and specific competencies for forensic nurses^(3)^.

Forensic nurses highlighted the origin and development of the specialty, connecting elements of academic training with professional practice to strengthen this area. Forensic sciences can be understood as a set of specialties that operate at the interface between clinical and legal issues, using knowledge from medicine, biology, social sciences, anthropology and criminalistics in the forensic field.

In nursing, this specialization arises from the broad field of forensic medicine and creates an approach that places nurses as a relevant partner in the judicial system. Forensic nursing encompasses the use of scientific and technical nursing knowledge in forensic clinical settings, uniting legal understanding with patient care. This integration represents significant progress in the care of victims of violence^(26)^.

The creation of appropriate spaces for carrying out forensic nursing actions takes into account cultural and national particularities, and is motivated by the type of care provided, which often includes patients in vulnerable conditions. This context is consistent with studies that highlight the role of nurses as the professionals who are at the forefront of care, often being the first to welcome victims of violence when they are treated in health units^(27)^.

Based on reports identified in this study, it was observed the importance of implementing forensic knowledge in already established environments and by nursing professionals who, on some occasions, do not use such knowledge and are not always properly prepared to deal with these situations, as many lack the necessary training to assist victims of violence^(4, 26)^. Many patients seeking medical care present clinical symptoms that may be indicative of violence. Therefore, training health professionals is essential to enable the identification of possible cases of violence through the assistance provided to users.

Therefore, it is essential to promote the training of professionals so that they are able to provide appropriate care to victims of violence who seek health services. These professionals need to be prepared to offer humane and welcoming care, both to victims and, in some situations, to aggressors^(27)^. When playing the role of health educator, nurses adopt a unique perspective in relation to human beings both individually and collectively^(27)^. This approach allows you to provide nursing care focused on health promotion and prevention, which goes beyond simply carrying out technical procedures.

Healthcare professionals must listen carefully, free from prejudice and judgment, valuing patients’ autonomy and establishing a relationship of trust. Comprehensive care is essential for the work of nurses who are dedicated to quality care, making qualified training absolutely necessary.

Professionals working in practical activities in the forensic area highlighted the relevance and necessity of their work. They also emphasized the urgency of improving public policies and developing teaching in this field at various academic levels, in line with continuing education in health, which seeks to reorient care practices. This approach is essential to transform work processes, based on the critical reflection of professionals about the reality of services and the collective search for solutions to identified problems^(27)^. Furthermore, the desire to develop the specialty was manifested in the difficulties mentioned for its consolidation and expansion of its possibilities. Although forensic nursing is gaining space in the global health context, there are still obstacles to be overcome for a more efficient organization of care^(27)^.

Throughout their training, forensic nurses develop specific competencies and knowledge that allow them to provide assistance in contexts of violence, and are recognized as experts with specialization in the area. Their work in forensic science is divided into two main areas: civil and criminal. In the scope of civil forensic science, they can perform audits of medical bills, management of health services and investigation of cases related to lack of competency, recklessness or negligence in nursing practice, including adverse events that may occur during care for users^(27)^.

The evolution of forensic nursing has shown its important contribution in the criminal and civil spheres, highlighting the urgent need for its recognition and application in forensic expertise in Brazil. Although studies on this field are still in their early stages, the literature highlights numerous opportunities for action, supported by scientific and humanistic foundations, which are crucial to clarify crimes, support victims and aggressors, and offer assistance to families. In the civil context, the role of nurses become especially significant, allowing for the performance of audits and specialized consultancy in nursing^(28)^.

Research on the topic, the dissemination of knowledge in the area and the growth in demand for specializations in forensic nursing are directly linked to the increase in violence in the world^(29)^. The increase in violent situations has created an urgent demand for training for nursing professionals and encouraged the search for preventive education focused on interpersonal violence as well as on identifying signs of vulnerability and victimization.

To strengthen the position of forensic nurses in Brazil, it is essential to establish a dialogue with the legislative branch with a view to creating standards that recognize forensic nurses as criminal experts. Furthermore, it is essential to widely publicize the relevance of this specialty for both society and public administration. It is also necessary to incorporate specific disciplines into undergraduate courses and to expand the offer of graduate courses with theoretical and practical content^(28)^.

Forensic nurses can play a key role in humanitarian forensics, a branch of forensic science focused on humanitarian goals. This specialty can be used to identify victims of large-scale disasters, people missing as a result of conflict, or victims of epidemic outbreaks^(30)^. However, for nurses to become forensic experts in Brazil, it is essential that there is a partnership with the legislative branch, with the aim of creating legislation that officially recognizes the role of forensic nurses as criminal experts^(28)^.

Among the limitations of this study, the small number of participants for a national study in the largest health category, which is nursing, stands out. However, it demonstrates the need to expand and disseminate this area of knowledge. Its strengths are the current characterization of the professionals involved and committed to an expanding area in nursing as well as presenting some tangible perspectives for the specialty. Committed professionals’ involvement enhances the establishment of development strategies for the specialty, in addition to subsidizing future actions for the application of forensic nursing in health services. Another highlight refers to the fact that this is the first national study with primary data on the characteristics of forensic nursing professionals a decade after the recognition of the specialty.

## CONCLUSION

This study allowed us to contextualize the current scenario of forensic nursing in Brazil. It is noteworthy that most participants were located in the Northeast and Southeast regions of the country, which shows greater development of the specialty in these regions. The emergence of the specialty occurred in the Northeast region, and its expansion occurred mainly with the support of the representative entities of forensic nursing in Brazil, which are SOBEF and ABEFORENSE, supported by COFEN.

The findings of this study reveal violence as the focus of the actions developed by the specialty. Forensic nursing uses scientific and legal knowledge to control violence and treat its victims. It is challenging for health service professionals to provide humanized care amidst so much violence and professional insecurity. Raising awareness about the implementation of forensic practices is essential to facilitate the action of justice in judicialized cases.

Finally, this study allowed us to relate forensic nursing experts’ knowledge and experiences, enabling us to understand the path already taken, as well as what still needs to be covered with political support through the development of public policies that include the practice of forensic nursing. The technical and scientific competencies already practiced by nursing must be exercised with a broader critical eye when faced with situations of violence, and the prospects for advancement in the area are related to the expansion of knowledge that will enable the rise of the specialty. There are still difficulties such as the lack of knowledge among professionals and the lack of political involvement to strengthen the specialty. Such difficulties can be justified by the territorial size of this country as well as by the cultural specificities of each region.
