# Supplementary material for: Brazilian forensic nursing from the perspective of its experts
Source: Rev Esc Enferm USP. 2025 May 2;59:e20240402. doi: 10.1590/1980-220X-REEUSP-2024-0402en (PMC12063539; doi:10.1590/1980-220X-REEUSP-2024-0402en)
Supplement: Translation

# Translation: A enfermagem forense brasileira na perspectiva de seus especialistas

**DOI:** 10.1590/1980-220X-REEUSP-2024-0402pt

**Authors:** Davydson Gouveia Santos, Vanessa Martinhago Borges Fernandes, Morgana Oliveira Citolin, Andreza Goulart Hilleshein, Maria de Fátima Saturnino, Mara Ambrosina de Oliveira Vargas

**Affiliations:** 1 Universidade Federal de Santa Catarina, Programa de Pós-Graduação em Enfermagem, Florianópolis, SC, Brasil.

**Keywords:** Enfermagem Forense, Cuidados de Enfermagem, Ciência e Desenvolvimento, Perfil de Competências de Enfermeiros, Enfermagem Prática

## Abstract

**Objetivo:**

Compreender a enfermagem forense brasileira na perspectiva de profissionais especialistas.

**Método:**

Estudo qualitativo, realizado com enfermeiros especialistas em enfermagem forense. A coleta de dados foi feita por meio de questionário eletrônico aplicado entre fevereiro e maio de 2023, composto por questões acerca de características socioprofissionais e de atuação. Os dados foram organizados por análise lexicográfica pelo IRAMUTEQ, analisados na perspectiva de análise de conteúdo de Bardin.

**Resultados:**

Participaram 55 especialistas que atuavam nas cinco regiões brasileiras, com maior participação das regiões Nordeste e Sudeste, ambas com 41,8% de profissionais. A maioria referiu ser mulher, branca, majoritariamente casada ou em união estável. A partir da associação das classes apresentadas no dendrograma gerado pelo IRAMUTEQ, foram geradas cinco categorias relacionadas aos termos lexicais da Classificação Hierárquica Descendente: 1) Criação da enfermagem forense e seu desenvolvimento político no território nacional; 2) Estabelecimento de ambientes para a atuação da enfermagem forense conforme a realidade nacional/cultural; 3) Competências já estabelecidas da enfermagem forense brasileira; 4) Caminhos para o sucesso da enfermagem forense brasileira; e 5) Perspectivas para a enfermagem forense brasileira.

**Conclusão:**

O estudo permitiu contextualizar o atual cenário da enfermagem forense brasileira. Os resultados revelaram a violência como foco das ações desenvolvidas pela especialidade. Por fim, foi possível relacionar o conhecimento e as experiências dos especialistas em enfermagem forense, viabilizando a compreensão da trajetória já percorrida e do que ainda precisa ser trilhado, como o desenvolvimento de políticas públicas que incluam o exercício do enfermeiro forense.

## INTRODUÇÃO

As ciências forenses são um conjunto de bases técnico-científicas aplicadas à investigação de crimes e situações de violência com uma abordagem voltada para o aspecto jurídico. O termo “ciência forense” passou a ser inserido nas ciências da saúde intencionalmente para capacitar profissionais em várias áreas, especialmente da enfermagem^(1)^.

No Brasil, a Resolução nº 389/2011 do Conselho Federal de Enfermagem (COFEN) reconhece a enfermagem forense como especialidade da categoria profissional entre as 65 especialidades existentes^(2)^. A Resolução n° 556/2017 do COFEN regulamentou a prática do enfermeiro forense brasileiro, suas áreas de atuação e as competências técnicas da especialidade^(3)^.

De acordo com essa resolução, o enfermeiro forense pode atuar em uma ampla gama de situações, incluindo trauma, violência, abuso sexual e uso de drogas, além de patologias psiquiátricas. Sua atuação abrange a assistência a diversos grupos, como agressores, populações vulneráveis e o sistema prisional. Inclui atividades como perícias e consultorias, além da atuação em desastres em massa e diferentes formas de violência, muitas vezes fora dos ambientes hospitalares. Para desempenhar essa função, o enfermeiro forense precisa possuir um título de especialização reconhecido pelo Ministério da Educação ou por instituições vinculadas no âmbito do Sistema COFEN/Conselhos Regionais^(3)^.

São competências fundamentais do enfermeiro forense a conservação dos vestígios e da cadeia de custódia, a obtenção de dados por meio de fotografias e documentação, assim como a elaboração de relatórios e pareceres para os tribunais. Ademais, tem a incumbência de oferecer consultoria em casos que envolvem a área forense, incluindo questões de saúde, lesões corporais, fraudes e outras formas de abuso^(4)^.

O trabalho dos enfermeiros forenses é bastante diversificado, crucial no atendimento às vítimas de violência, pois normalmente o profissional de enfermagem presta o primeiro suporte ao paciente em um ambiente hospitalar^(4)^. Ademais, a violência tem aumentado progressivamente com o passar dos anos, refletindo uma complexa interação de fatores sociais, econômicos, políticos e culturais^(5)^. Como um fenômeno sistêmico, a violência influencia de maneira direta os sistemas de saúde, segurança e previdência, com impacto negativo na qualidade de vida da população.

O desenvolvimento da enfermagem forense no Brasil pode contribuir de forma significativa para aprimorar a qualidade de vida das vítimas e ajudar na prevenção de episódios de violência^(6)^. O enfermeiro tem papel essencial no cuidado das vítimas de violência, sendo indispensável na anamnese, na realização do exame físico e no diagnóstico de enfermagem. Essas etapas são determinantes na entrada no sistema de saúde^(7)^. Independentemente do tempo de experiência na área da saúde, os enfermeiros reconhecem a importância da formação forense no contexto clínico, para garantir o cumprimento dos princípios éticos e legais e assumir a responsabilidade pelo reconhecimento, coleta e preservação de provas no contexto da assistência ao usuário com necessidades físicas, psicológicas e psicossociais complexas^(8, 9)^.

Tendo em conta o papel crucial do enfermeiro no atendimento e no suporte ao paciente, ele se torna figura central no processo de assistência às vítimas de violência dentro do contexto de cuidado. A enfermagem forense diferencia-se por unir o sistema de saúde ao sistema judicial, envolvendo ações como buscas, investigações e cuidados. Além da prática clínica, essa área da enfermagem desempenha um papel importante na coleta de evidências, ao se envolver ativamente na prevenção, identificação e combate à violência, contribuindo para a elaboração de respostas a diferentes situações de agressão^(10)^.

Ao aplicar conhecimentos de enfermagem forense, o enfermeiro pode identificar casos de violência e promover atendimento mais humanizado, já que suas ações podem ajudar a romper o ciclo da violência. Para isso, precisam ser adaptadas às necessidades individuais, fundamentadas em evidências científicas e elaboradas conforme as atuais leis, políticas públicas de saúde e recursos básicos da enfermagem, com o objetivo de reduzir os danos causados às vítimas de violência^(11)^.

Diante do exposto, e por se tratar de uma área emergente ainda não incorporada às instituições de saúde brasileiras, o objetivo deste artigo foi compreender a enfermagem forense brasileira na perspectiva de profissionais especialistas. Para isso, foram considerados os aspectos históricos, a realidade atual e as perspectivas de avanço para especialidade.

## MÉTODO

### Tipo do Estudo

Estudo descritivo, com abordagem exploratória e de natureza qualitativa, A apresentação seguiu as recomendações do *COnsolidated criteria for REporting Qualitative research* (COREQ)^(12)^.

### Local

A amostragem por conveniência foi empregada, e os dados foram coletados por meio da distribuição de questionário *online* em plataformas virtuais, como *e-mail, Instagram* ^®^ *, Facebook* ^®^ e grupos do *WhatsApp* ^®^. O questionário foi disponibilizado no *Google Forms* ^®^, e essa abordagem permitiu alcançar todas as regiões do Brasil.

### População e Critérios de Seleção

A população do estudo foi composta por enfermeiros forenses com experiência prática assistencial ou docência em território nacional, o que incluiu pessoas de diferentes regiões do país e permitiu a compreensão das variações regionais.

Os critérios de inclusão para participar do estudo foram ser enfermeiros forenses com especialização *lato sensu* em enfermagem forense ou *expertise* na área, com experiência prática assistencial ou de docência em território nacional. O critério de exclusão abrangeu os profissionais que possuíam interesse na área, mas não possuíam habilitação teórica e prática relacionada à enfermagem forense.

### Definição da Amostra

A técnica *snowball*, uma amostragem não probabilística, foi empregada para o recrutamento dos participantes. As cadeias de referência foram utilizadas para localizar potenciais participantes. Inicialmente, os primeiros indivíduos foram convidados por meio de indicação do primeiro autor deste estudo e, a seguir, por sugestões dos próprios participantes. A coleta de dados foi interrompida quando as indicações começaram a se repetir, sem respostas adicionais ao questionário *online*. No total, 63 respostas foram registradas, e após refinamento dos dados, 55 enfermeiros forenses atenderam aos critérios de inclusão e completaram o questionário.

### Coleta de Dados

Os dados foram coletados entre fevereiro e maio de 2023, por meio de questionário estruturado desenvolvido pelos autores, que foi dividido em quatro partes. A primeira parte continha o título da pesquisa, o convite para participação, os critérios de inclusão, o Termo de Consentimento Livre e Esclarecido, e as opções “Li os termos e concordo em participar da pesquisa” e “Li os termos e não concordo em participar da pesquisa”. Ao selecionar a opção de concordância, o participante tinha acesso ao questionário, enquanto, caso contrário, o convite era encerrado e o *Google Forms* ^®^ redirecionava-o para uma página de agradecimento. A segunda parte abordava questões sobre as características dos participantes. A terceira parte aplicava a Técnica de Associação Livre de Palavras (TALP), que solicitava aos participantes que mencionassem as cinco palavras que lhes vinham à mente ao pensar sobre a atuação do enfermeiro forense no Brasil. A TALP é uma técnica subjetiva usada para explorar conteúdos latentes não filtrados por censura. A quarta parte tratava da aplicação da enfermagem forense no Brasil.

### Análise e Tratamento dos Dados

Os dados do perfil socioprofissional foram registrados em planilha do *Microsoft Excel* ^®^, com dupla conferência, e processados pelo *software Statistical Package for the Social Sciences* (SPSS), versão 29. A análise seguiu a estatística descritiva, com medidas de tendência central (média, mediana, máximo e mínimo) e dispersão (desvio padrão). Os dados obtidos por meio da TALP e sobre o desenvolvimento da enfermagem forense no brasil foram analisados lexicograficamente, utilizando o *software Interface de R pour les Analyses Multidimensionnelles de Textes et de Questionnaires* (IRAMUTEQ).

O IRAMUTEQ^(13)^ é uma ferramenta que organiza textos com base na semelhança entre os vocábulos, permitindo a criação de classes e categorias que agrupam os termos mais significativos. Esses resultados são apresentados em forma de nuvem de palavras e dendrograma, com a utilização da Classificação Hierárquica Descendente (CHD). Para interpretar os dados da CHD, foram analisadas formas ativas (adjetivos, advérbios, substantivos, verbos) de cada classe de segmentos. Textos com valor ≥ 3,26 e valor de p ≤ 0,03433 no teste qui-quadrado (chi^2^) indicam significância estatística na associação das palavras dentro de sua classe.

Após o processamento, o núcleo de sentido dos segmentos foi analisado, destacando as palavras mais significativas. Isso possibilitou a categorização dos conteúdos léxicos nas respostas, organizadas por eixos temáticos mediante análise de conteúdo. Esse tipo de análise envolve a leitura das evidências produzidas, a codificação das unidades de significado, a interpretação e a formulação de inferências, além da análise das categorias temáticas geradas pelo IRAMUTEQ, estabelecendo conexões com os marcos conceituais da pesquisa.

Os dados da quarta etapa foram analisados por meio da técnica de análise de conteúdo proposto por Bardin^(14)^, com apoio do *software Qualitativa Data Analysis* (QDA) *Miner* ^(15)^. A análise de conteúdo compreende o “conjunto de técnicas da análise das comunicações”, e envolve as etapas de pré-análise; exploração do material; tratamento dos dados e interpretação.

Na pré-análise, os dados foram transportados para o QDA *Miner*, sendo realizadas leituras flutuantes para conhecer o texto e selecionar indicadores para a fase seguinte. Na exploração do material, foram realizadas a codificação e condensação das unidades de registro para a construção das categorias empíricas. Em seguida, foram realizados os tratamentos dos dados e a interpretação, de acordo com a leitura sobre o tema.

### Aspectos Éticos

O estudo foi aprovado pelo Comitê de Ética em Pesquisa da Universidade Federal de Santa Catarina, sob Parecer n^o^ 5.808.287. Todos os participantes assinaram o Termo de Consentimento Livre e Esclarecido na modalidade *online*, por meio da seleção da opção “Li os termos e concordo em participar da pesquisa”. Todos os aspectos éticos de pesquisas que envolvem seres humanos foram atendidos. Para garantir o anonimato, os participantes foram identificados pela abreviatura “EF” (enfermeiro forense), seguida de um número cardinal em ordem crescente.

## RESULTADOS

Participaram do estudo 55 (100%) enfermeiros, sendo 46 (83,6%) do sexo feminino e nove (16,4%) do masculino, com média de idade de 41 anos (DP = 10,5; Mín = 23; Máx = 65). Quanto à cor, 29 (52,7%) autodeclararam-se brancos, 18 (32,7%), pardos, sete (12,7%), negros, e um (1,8%), amarelo. Eram majoritariamente casados ou em união estável (n = 26/47,3%). Além disso, 22 (40%) eram solteiros, seis (10,9%), divorciados, e um (1,8%), viúvo. A religião predominante foi o catolicismo, citado por 30 (54,5%) participantes; seis (10,9%) declararam-se protestantes ou evangélicos; cinco (9,1%) declararam-se agnósticos; quatro (7,3%) declararam-se espiritas/espiritualistas; seis (10,92%) professavam outras crenças cristãs ou de matriz africana (cristão, testemunha de jeová, candomblé, umbanda); e quatro (7,2%) declararam-se ateus ou sem crença religiosa.

Em relação ao tempo de graduados, 32 (58,2%) relataram entre seis e 20 anos de atuação profissional, 18 (32,7%), entre 21 e 40 anos, e apenas cinco (9,1%), até cinco anos. Além disso, 14 (25,5%) possuíam formação completa de doutorado, dez (18,2%), de mestrado, e 31 (56,3%), de especialização. O perfil de formação desses profissionais é apresentado na Tabela 1.

**Tabela 1:** Perfil da formação em enfermagem ao aperfeiçoamento em enfermeiros forenses Florianópolis, SC, Brasil, 2024.

Variáveis		N	%
**Tempo de formação em enfermagem**	Até cinco anos de formação	5	9,1
	Seis a dez anos de formação	12	21,8
	11 a 20 anos de formação	20	36,4
	21 a 30 anos de formação	11	20
	Acima de 30 anos de formação	7	12,7
	Total	55	100
**Maior titulação**	Doutorado	14	25,5
	Mestre	10	18,2
	Especialista	31	56,3
	Total	55	100
**Ano da formação em enfermagem forense**	2015	2	3,6
	2016	1	1,8
	2018	9	16,4
	2019	16	29,1
	2020	2	3,6
	2021	8	14,5
	2022	9	16,4
	2023	3	5,5
	2024	5	9,1
	Total	55	100
**Como adquiriu o título de especialista em enfermagem forense**	Especialização presencial	19	34,5
	Especialização a Distância (EAD)	11	20
	Prova de títulos das entidades de classe representativas da área	20	36,4
	Cursando + cursos livres	5	9,1
	Total	55	100
**Instituição que conferiu o título de enfermeiro forense**	ABEFORENSE	8	14,5
	SOBEF	11	20
	IDE – Universidade Cristo Redentor	8	14,5
	Instituto Keynes	6	10,9
	UNIFAJ	5	9,1
	Unyleya	4	7,3
	Outros	13	23,7
	Total	55	100
**Atuação profissional por região nacional**	Região Centro-Oeste	3	5,5
	Região Nordeste	23	41,8
	Região Norte	1	1,8
	Região Sudeste	23	41,8
	Região Sul	5	9,1
	Total	55	100

Fonte: dados da pesquisa. 2023.

Entre os participantes do estudo, 28 (50,9%) tinham registro de especialista em enfermagem forense no COREN ao qual estavam vinculados. Quanto à vinculação às entidades representativas da área, 20 (36,4%) estavam associados à SOBEF, 12 (21,8%), à ABEFORENSE, e 23 (41,8%) não estavam vinculados aos órgãos representativos da área.

A TALP deu-se pela citação de cinco palavras ao pensar a respeito da “atuação do enfermeiro forense no Brasil”. Os cognemas evocados foram registrados na ordem de menção. Foram evocadas 275 palavras/expressões por 55 enfermeiros, e foram identificadas 29 palavras-expressões distintas com maior frequência. O *corpus* das evocações foi analisado no *software* IRAMUTEQ, utilizando a técnica de nuvem de palavras, que é um dos métodos de processamento de dados aplicados neste estudo. Essa abordagem permite a apresentação gráfica das palavras mais frequentes, com o tamanho de cada termo sendo proporcional ao número de vezes em que foi mencionado. Entre as palavras-chave de maior recorrência durante a coleta, destacou-se o termo “violência”, citado por 21 dos participantes. Termos citados menos de duas vezes foram excluídos do gráfico. Assim, gerou-se a Figura 1.

**Figura 1:** Nuvem de palavras gerada pela Técnica de Associação Livre de Palavras acerca da atuação da enfermagem forense no Brasil. Brasil, 2023.

Fonte: *software* IRAMUTEQ, 2023.

A nuvem de palavras foi utilizada como ponto de partida para a análise dos dados. As palavras com menor destaque indicam diferentes aspectos que influenciam o cenário da enfermagem forense, estabelecendo conexões diretas com os termos mais destacados. Entre os termos mais recorrentes, destacam-se “violência” (21), “cuidado” (11), “desafio” (oito), além de “justiça”, “humanização” e “vestígio”, que foram mencionados sete vezes cada. A CHD possibilitou a segmentação do *corpus* textual em classes de segmentos de texto (STs) e seus respectivos vocábulos, evidenciando as ideias centrais presentes nas respostas dos participantes. A análise hierárquica resultou em 151 STs, dos quais 132 foram classificados, obtendo-se um aproveitamento de 87,42% e formando cinco classes. Ou seja, criaram-se cinco agrupamentos de STs com vocábulos semelhantes e correlacionados entre si, mas distintos dos demais.

No dendrograma, o *corpus* textual foi dividido em dois grupos: o primeiro, composto pelas classes II e III, representando, respectivamente, representando 18,2% e 15,2% dos STs; o segundo grupo, formado por uma subdivisão da classe V, correspondendo a 23,5% dos STs, originando as classes I e IV, que representam 18,9% e 24,2% dos STs, respectivamente (Figura 2).

**Figura 2:** Dendrograma da Classificação Hierárquica Descendente com palavras significativas acerca do desenvolvimento da enfermagem forense brasileira. Brasil, 2023.

Fonte: elaborado pelos autores, a partir dos dados gerados pelo *software* IRAMUTEQ, 2023.

Diante do processamento do *corpus* pelo *software*, verificou-se que as classes I, IV e V estão associadas entre si e opõem-se, em termos lexicais, às classes II e III. Os STs de cada *cluster* foram resgatados e interpretados de forma detalhada, tendo como base os princípios da análise temática, visando compreender seus núcleos de sentido e assim categorizando as classes. A partir dos resultados apresentados no dendrograma da CHD, as classes categorizadas foram descritas e exemplificadas com excertos extraídos do questionário.

A classe I (Criação da enfermagem forense e seu desenvolvimento político no território nacional), relativa aos marcos de criação e desenvolvimento da especialidade no Brasil, reuniu 25 STs (18,9%) do *corpus* total analisado, constituída por palavras e radicais no intervalo entre x^2^ = 4,55 (crescimento) e x^2^ = 36,45 (Brasil), como “criação” (x^2^ = 17,65), “elaboração” (x^2^ = 13,14) e “especialidade” (x^2^ = 5,84).

Os enfermeiros forenses mencionaram o surgimento e o desenvolvimento da especialidade, e relacionaram elementos da formação com a prática profissional para o fortalecimento da área: [...] *Resolução no 556/2017 do COFEN* (EF32); *Elaboração de protocolos e quesitos judiciais* (EF03); [...] *criação de projetos para fomentar a criação de cargos de enfermeiros forenses em Minas Gerais* (EF20); [...] *uma vez que com o aumento de pós-graduações o número de especialistas torna-se maior e faz com que a área se fortaleça* (EF50); [...] *o profissional da enfermagem é o primeiro profissional a abordar o paciente na unidade hospitalar; logo, tem muito a contribuir para a coleta de vestígios e informações a serem entregues à justiça* (EF16); *Eu ingressei na pós-graduação e tenho me encantado com o mundo da* [enfermagem] *forense e com a potência dessa especialidade; procuro colocá-la em tudo o que eu consigo* (EF17); [...] *tenho certeza de que haverá um antes e depois na sociedade brasileira após a criação do cargo e da atuação dos enfermeiros forenses na área* (EF42).

A classe II (Estabelecimento de ambientes para a atuação da enfermagem forense conforme a realidade nacional/cultural) diz respeito aos possíveis atendimentos da especialidade, por vezes caracterizados pela fragilidade dos atendidos. Abrangeu 24 STs (18,2%) do *corpus* total analisado, sendo constituída por palavras e radicais no intervalo entre x^2^ = 4,48 (reconhecer) e x^2^ = 27,5 (âmbito), como “contexto” (x^2^ = 24), “lesão” (x^2^ = 18,56), “identificar” (x^2^ = 13,81) e “intervenção” (x^2^ = 6,11).

A análise revelou a necessidade da aplicação do conhecimento forense em ambientes já existentes pelos profissionais de enfermagem, que por vezes não aplicam cuidados forenses: [...] *prestar apoio de consultoria em casos de litígios relacionados à área forense no âmbito de cuidados em saúde, responsabilidade civil por lesões corporais e outros abusos;* [...] *executar atividades de enfermagem forense prisional com ofensores, penalizados judicialmente, focando numa abordagem interdisciplinar na tomada de decisões com o sistema judiciário e de segurança pública, no âmbito do direito penal, civil, militar e do trabalho, com objetivo de ressocialização;* [...] *coletar, recolher e preservar vestígios, na vítima e no perpetrador, nos diferentes contextos da prática de enfermagem forense, em âmbito pré-hospitalar, hospitalar, comunitário, em Unidade Básicas de Saúde Prisional* (UBSP) *ou outros contextos profissionais, com observância dos limites legais aplicáveis;* [...] *participar de medidas de preservação de cadáveres no âmbito de desastres em massa, rebeliões, catástrofes e missões humanitárias;* [...] *realizar acolhimento de vítimas e potenciais vítimas em situações de violência sexual, executar cuidados de enfermagem no âmbito forense e reconhecer a importância da participação da vítima na coleta, recolha e preservação de vestígios* [...] (EF18); *Falta conhecimento das pessoas acerca da enfermagem forense. Há necessidade de criação de empregos formais para atuar como enfermeiro forense, bem como elaboração e divulgação da enfermagem forense como ciência e atuação como profissional da saúde nos diversos ambientes onde sua atuação se faz necessária* (EF29); e *Alcançar ambientes profissionais* (EF43).

A classe III (Competências já estabelecidas da enfermagem forense brasileira) apresenta os elementos que já constam das atuais competências, mas é preciso ir além, ampliando-as de acordo com possibilidades que a área possui. Reuniu 20 STs (15,2%) do *corpus* total analisado, e foi constituída por palavras e radicais no intervalo entre x^2^ = 6,34 (coleta) e x^2^ = 35,2 (vestígio), como “informação” (x^2^ = 29,1), “proteção” (x^2^ = 23,1) e “perpetrador” (x^2^ = 13,98).

As competências podem ser ampliadas conforme acontece a disseminação da área e as qualificações dos profissionais atuantes nos serviços que prestam atendimento às vítimas e aos perpetradores: *Quando iniciei os estudos da especialização* (enfermagem forense), *elenquei as competências do enfermeiro forense e acrescentei algumas: aplicar os processos de enfermagem no atendimento à população prisional na prevenção dos maus-tratos, violência sexual e outras formas de violência;* [...] *prestar apoio de consultoria em casos de litígios relacionados à área forense no âmbito de cuidados em saúde, responsabilidade civil por lesões corporais e outros abusos; Emitir pareceres informativos, enquanto consultor forense, sobre a prestação de cuidados de saúde e os resultados pertinentes;* [...] *validar as coletas de vestígios feitas por enfermeiros forenses* (EF18); [...] *educação dos pacientes e familiares para o reconhecimento das expressões da violência e acionamento da rede de suporte; acionar a equipe interprofissional para proteção e advocacia dos pacientes avaliados; educação para comunicação não violenta; formação de grupos de apoio e grupos reflexivos no trabalho baseado em gênero, sexualidade e masculinidade para o enfrentamento da violência* (EF5); *Acolhimento e assistência de enfermagem a vítimas/perpetradores/famílias; encaminhamentos à rede de proteção; coleta e preservação de vestígios e exames; avaliação e registro de relatórios periciais; intervenções e treinamentos de profissionais e população em geral para prevenção da violência* (EF34).

A classe IV (Caminhos para o sucesso da enfermagem forense brasileira) reúne as ações que, ao serem implantadas, podem favorecer a consolidação da especialidade do Brasil. Foi composta por 32 STs (24,2%) do *corpus* total analisado, constituída por palavras e radicais no intervalo entre x^2^ = 5,79 (SOBEF) e x^2^ = 23,1 (tema), tais como “reconhecimento” (x^2^ = 21,98), “institucional” (x^2^ = 12,89), “políticas públicas” (x^2^ = 9,59) e “científico” (x^2^ = 9,59).

Os profissionais que exercem atividades práticas relacionadas à área forense ressaltaram sua importância, além da necessidade de fortalecimento de políticas públicas e avanço do ensino nos diferentes níveis acadêmicos: *Reconhecimento na área da pesquisa/acadêmica* (EF2); *Reconhecimento da importância da aplicação da expertise forense nos contextos de saúde coletiva* (EF4); *Reconhecimento profissional por meio da elucidação de casos* (EF18); *Reconhecimento frente aos demais colegas nas ciências forenses* (EF30); *Reconhecimento social e institucional; políticas públicas; instituições de ensino qualificadas para treinamentos e capacitação; validação de protocolos seguindo o rigor devido; articulação de serviços e profissionais* (EF34); *Ser reconhecida nos organogramas dos serviços assistenciais e ter mais protagonismo na área da violência* (EF54).

A classe V (Perspectivas para a enfermagem forense brasileira) reflete o desejo positivo de evolução para a especialidade, assim como as dificuldades dos profissionais para sua consolidação. Composta por 31 STs (23,5%) do *corpus* total analisado, foi constituída por palavras e radicais no intervalo entre x^2^ = 4,66 (especialização) e x^2^ = 15,93 (precisar), tais como “momento” (x^2^ = 15,84), “conhecimento” (x^2^ = 12,71), “trabalhar” (x^2^ = 10) e “profissional” (x^2^ = 9,18).

O desejo de ascensão da especialidade foi expresso pelas dificuldades que a área tem enfrentado, mas também por suas potencialidades: *A enfermagem forense tem um potencial enorme para crescimento e atuação no Brasil devido à necessidade de acolhimento e assistência às vítimas de violência tão presentes em nosso país* (EF29); *Sinceramente, são pequenas. Devido ao que falei acima, infelizmente, o Brasil é um país congelado em seus princípios e tem que ter muita luta para se conseguir algo, mas gostaria de acreditar que um dia seremos iguais aos EUA e ter uma enfermagem forense realmente atuante* (EF17); *Conseguir contato de profissionais com conhecimento na área e expandir a profissão em nosso estado de Santa Catarina. Atualmente, a enfermagem forense concentra-se com a maioria dos profissionais mais conceituados no nordeste do país, e precisamos inserir enfermeiros forenses em toda a rede e instituições de saúde* (EF37); *Muito baixa, pois não vejo políticas públicas ou autoridades brigando pela nossa profissão* (EF24); e *Reconhecimento da especialidade como caminho para combate à violência e inserção de políticas públicas contemplando a enfermagem forense* (EF47).

## DISCUSSÃO

O presente estudo compreendeu o processo de inserção da enfermagem forense no cenário brasileiro, os ambientes em que as práticas da especialidade podem e devem ser exercidas, assim como as possibilidades de ampliação das competências estabelecidas, os caminhos que a área precisa trilhar para a consolidação nacional e as perspectivas dos profissionais que estão relacionadas à superação dos desafios existentes para aplicação do conhecimento.

Os cuidados de enfermagem forense envolvem a aplicação de habilidades práticas e pensamento crítico acerca do processo de enfermagem para impactar positivamente a saúde do paciente afetado por trauma ou pela violência, por meio de identificação e anotações de evidências forenses que devem ser apresentadas à justiça criminal sobre os cuidados prestados ao indivíduo^(16)^.

Investigações acerca das práticas da enfermagem forense são desenvolvidas mundialmente^(16)^. Em território nacional, estudos vêm sendo desenvolvidos relacionando as possibilidades de execução das práticas forenses durante os atendimentos já prestados por enfermeiros generalistas e especialistas em outras áreas, como emergência, obstetrícia, violência sexual, sistema prisional^(9, 19, 20)^, entre outros. Há necessidade da inserção dos especialistas nos serviços especializados ao atendimento de pessoas em situações vulneráveis ou de violência, como também os perpetradores dessas situações.

Este estudo converge com outros sobre a importância de promover a conscientização sobre a enfermagem forense, regulamentada recentemente no Brasil, assim como suas diferentes áreas de atuação^(9, 21)^. Embora a formação específica nesse setor ainda seja limitada, os profissionais de enfermagem prestam cuidados assistenciais de acordo com as diretrizes estabelecidas, frequentemente guiados por protocolos institucionais. Isso evidencia a estratégia utilizada no atendimento a vítimas de violência, e o enfermeiro exerce a função de acolhimento e orientação do paciente para outras especialidades^(19)^.

A violência encontra-se no centro do trabalho do enfermeiro forense, seja na prevenção, no controle ou no tratamento das pessoas vítimas ou perpetradores de violência. Classicamente, a violência é designada pela Organização Mundial da Saúde como um grave problema de saúde pública que pode ser evitável. É definida como “o uso intencional da força física ou do poder, real ou em ameaça, contra si próprio, contra outra pessoa, ou contra um grupo ou uma comunidade, que resulte ou tenha grande possibilidade de resultar em lesão, morte, dano psicológico, deficiência de desenvolvimento ou privação”^(21)^. A realização do cuidado a ser prestado torna-se um desafio para os profissionais de enfermagem forense.

A enfermagem forense caracteriza-se como uma nova especialidade que vincula o saber científico, destacando-se em investigações de criminalidades, mas é preciso ter conhecimento científico para cumprir com êxito essa responsabilidade. É importante que o profissional tenha conhecimento adequado e sensibilidade para identificar a violência e, por meio de sua intervenção, ajudar na promoção da justiça^(22)^. Os enfermeiros na linha de frente da assistência prestam os primeiros cuidados às vítimas de violência, independentemente de tempo de atuação no setor ou se se considerarem aptos ou preparados para esse tipo de atendimento. Além disso, existe escassez de recursos humanos e falta de reconhecimento sobre o papel da enfermagem forense entre os desafios do exercício da enfermagem^(23)^. A percepção dos enfermeiros que realizam o recolhimento, a identificação e a preservação de vestígios no atendimento a pacientes em situação de violência facilitam os trâmites legais dos casos que são judicializados.

A enfermagem forense resulta da junção da ciência de enfermagem com os sistemas de saúde e justiça. Sua trajetória teve início na década de 1970 nos Estados Unidos, quando um grupo de enfermeiras que defendia os direitos das mulheres lutava por um atendimento mais completo às vítimas de estupro, o que incluía a coleta de evidências médicas. As enfermeiras realizavam exames físicos e coletavam vestígios forenses durante o atendimento a vítimas de violência sexual, mas não eram reconhecidas como peritas e não tinham permissão para fornecer depoimentos às autoridades judiciais nos tribunais^(23)^.

Com a introdução do programa *Sexual Assault Medical Forensic Exam* nos Estados Unidos, na década de 1980, as enfermeiras foram reconhecidas como examinadoras, estabelecendo-se um novo campo de especialização para a profissão^(24)^. Diante da crescente necessidade de atendimento às vítimas de violência, foi imprescindível que as enfermeiras desenvolvessem novas habilidades e obtivessem certificações específicas para atuar no campo forense. Assim, passaram a atuar como examinadores independentes em equipes compostas por diversos especialistas, incluindo policiais, promotores e defensores públicos. Esse modelo pioneiro teve sua origem na Califórnia, nos Estados Unidos, por meio dos programas *Sexual Assault Nurse Examiner* e *Sexual Assault Response Teams* ^(25)^.

A formação e o avanço da especialidade de enfermagem forense no Brasil representam marcos importantes. Com maior progresso observado em nações como Portugal, Estados Unidos e Japão, a especialidade está em estágio inicial na América Latina e ainda é incipiente no país. No entanto, já é oficialmente reconhecida como uma especialização para enfermeiros pelo COFEN, conforme estabelecido pela Resolução nº 389/2011, mais tarde substituída pela Resolução nº 581/2018 do COFEN^(2)^. A enfermagem forense brasileira é regulamentada pela Resolução nº 556/2017, que define as diretrizes para a prática. Esta resolução estabelece as áreas de atuação, bem como as competências gerais e específicas para os enfermeiros forenses^(3)^.

Os enfermeiros forenses ressaltaram a origem e o desenvolvimento da especialidade, conectando elementos da formação acadêmica com a atuação profissional para fortalecer essa área. As ciências forenses podem ser entendidas como um conjunto de especialidades que operam na interface entre questões clínicas e legais, utilizando conhecimentos de medicina, biologia, ciências sociais, antropologia e criminalística no âmbito pericial.

Na enfermagem, essa especialização surge do amplo domínio da medicina forense, e cria uma abordagem que coloca o enfermeiro como um parceiro relevante no sistema judiciário. A enfermagem forense abrange a utilização de conhecimentos científicos e técnicos da enfermagem em contextos clínicos forenses, unindo o entendimento legal ao atendimento ao paciente. Essa integração constitui um progresso significativo para a assistência às vítimas de violência^(26)^.

A criação de espaços adequados para a realização de ações da enfermagem forense leva em consideração as particularidades culturais e nacionais, e é motivada pelo tipo de atendimento prestado, que frequentemente inclui pacientes em condições de vulnerabilidade. Esse contexto coaduna-se com estudos que ressaltam o papel do enfermeiro como o profissional que está na vanguarda do cuidado, muitas vezes sendo o primeiro a acolher as vítimas de violência ao serem atendidas nas unidades de saúde^(27)^.

A partir de relatos identificados neste estudo, observou-se a importância de implementar o conhecimento forense em ambientes já estabelecidos e por parte dos profissionais de enfermagem que, em algumas ocasiões, não utilizam tais conhecimentos e nem sempre estão devidamente preparados para lidar com essas situações, pois muitos carecem da formação necessária para atender vítimas de violência^(4, 26)^. Diversos pacientes em busca de atendimento médico apresentam sintomas clínicos que podem ser indicativos de violência. Assim, a capacitação dos profissionais de saúde é essencial para permitir a identificação de possíveis casos de violência por meio da assistência prestada aos usuários.

Assim, é fundamental promover a capacitação de profissionais para que sejam aptos a prestar um atendimento apropriado às vítimas de violência que buscam os serviços de saúde. Esses profissionais precisam estar preparados para oferecer um cuidado humanizado e acolhedor, tanto para as vítimas quanto, em algumas situações, quanto para os agressores^(27)^. Ao desempenhar o papel de educador em saúde, o enfermeiro adota uma perspectiva singular em relação ao ser humano tanto no âmbito individual quanto no coletivo^(27)^. Essa abordagem permite que preste cuidados de enfermagem voltados para a promoção e prevenção da saúde, que vão além da simples realização de procedimentos técnicos.

Os profissionais de saúde devem realizar uma escuta cuidadosa, isenta de preconceitos e julgamentos, valorizando a autonomia dos pacientes e estabelecendo uma relação de confiança. O cuidado integral é essencial para a atuação de enfermeiros que se dedicam à qualidade do cuidado, tornando absolutamente necessária a formação qualificada.

Os profissionais que atuam em atividades práticas na área forense destacaram a relevância e a necessidade de seu trabalho. Enfatizam também a urgência de aprimorar políticas públicas e fazer evoluir o ensino nesse campo em diversos níveis acadêmicos, em consonância com a educação permanente em saúde, que busca reorientar as práticas de cuidado. Essa abordagem é fundamental para transformar os processos de trabalho, fundamentada na reflexão crítica dos profissionais acerca da realidade dos serviços e na busca coletiva por soluções para os problemas identificados^(27)^. Além disso, o anseio por desenvolver a especialidade manifestou-se nas dificuldades mencionadas para sua consolidação e ampliação de suas possibilidades. Apesar de a enfermagem forense estar conquistando espaço no contexto global da saúde, ainda há obstáculos a serem enfrentados para uma organização mais eficiente dos cuidados^(27)^.

Ao longo de sua formação, o enfermeiro forense desenvolve habilidades e conhecimentos específicos que lhe permitem prestar assistência em contextos de violência, sendo reconhecido como um perito com especialização na área. Sua atuação na perícia divide-se em duas áreas principais: civil e criminal. No âmbito da perícia civil, pode realizar auditoria de contas médicas, gestão dos serviços de saúde e investigação de casos relacionados à falta de competência, imprudência ou negligência na prática da enfermagem, incluindo os eventos adversos que podem ocorrer durante o atendimento a usuários^(27)^.

A evolução da enfermagem forense tem mostrado sua importante contribuição nas esferas criminal e cível, ressaltando a necessidade urgente de seu reconhecimento e aplicação nas perícias forenses no Brasil. Apesar de os estudos sobre esse campo ainda estarem em estágios iniciais, a literatura evidencia inúmeras oportunidades de atuação, sustentadas por fundamentos científicos e humanísticos, que são cruciais para esclarecer crimes, apoiar vítimas e agressores, além de oferecer assistência às famílias. No contexto cível, a função do enfermeiro torna-se especialmente significativa, permitindo a realização de auditorias e consultorias especializadas na área da enfermagem^(28)^.

A pesquisa sobre o tema, a difusão do conhecimento na área e o crescimento da procura por especializações em enfermagem forense estão diretamente ligados ao aumento da violência no mundo^(29)^. O crescimento de situações violentas tem criado demanda urgente por formação para os profissionais de enfermagem e incentivado a busca por educação preventiva focada na violência interpessoal, bem como na identificação de sinais de vulnerabilidade e vitimização.

Para fortalecer a posição do enfermeiro especialista em perícia no Brasil, é fundamental estabelecer um diálogo com o poder legislativo visando à criação de normas que reconheçam o profissional enfermeiro forense como perito criminal. Ademais, é imprescindível a ampla divulgação sobre a relevância dessa especialidade tanto para a sociedade quanto para a administração pública. Também é necessário incorporar disciplinas específicas nos cursos de graduação e ampliar a oferta de pós-graduações com conteúdo teóricos e práticos^(28)^.

Os enfermeiros forenses podem exercer uma função fundamental na perícia humanitária, uma área da ciência forense voltada para objetivos humanitários. Essa especialidade pode ser empregada na identificação de vítimas de desastres em larga escala, pessoas desaparecidas em decorrência de conflitos ou vítimas de surtos epidêmicos^(30)^. Entretanto, para que o enfermeiro se torne um especialista em perícia no Brasil, é fundamental que ocorra uma parceria com o poder legislativo, com o intuito de criar legislações que reconheçam oficialmente a função do enfermeiro forense como perito criminal^(28)^.

Entre as limitações do presente estudo, destaca-se o número reduzido de participantes para um estudo nacional na maior categoria da saúde, que é a enfermagem. No entanto, demonstra a necessidade de ampliação e divulgação dessa área de conhecimento. Seus pontos fortes são a caracterização atual dos profissionais envolvidos e comprometidos com uma área em expansão na enfermagem, assim como apresenta algumas perspectivas palpáveis para a especialidade. O envolvimento de profissionais comprometidos potencializa o estabelecimento de estratégias de desenvolvimento para especialidade, além de subsidiar futuras ações para aplicação da enfermagem forense nos serviços de saúde. Outro destaque refere-se ao fato de este ser o primeiro estudo nacional com dados primários de características dos profissionais de enfermagem forense uma década após o reconhecimento da especialidade.

## CONCLUSÃO

Este estudo permitiu contextualizar o atual cenário da enfermagem forense brasileira. Destaca-se que a maioria dos participantes estava inserida nas regiões Nordeste e Sudeste do país, o que evidencia maior desenvolvimento da especialidade nessas regiões. O surgimento da especialidade ocorreu na região Nordeste, e sua expansão aconteceu principalmente com o apoio das entidades representativas da enfermagem forense no Brasil, que são a SOBEF e ABEFORENSE, apoiadas pelo COFEN.

Os achados deste estudo revelam a violência como foco das ações desenvolvidas pela especialidade. A enfermagem forense utiliza-se dos conhecimentos científicos e legais para o controle da violência e o tratamento de suas vítimas. Torna-se desafiador para os profissionais dos serviços de saúde desenvolver um cuidado humanizado em meio a tanta violência e insegurança profissional. A sensibilização para a execução das práticas forenses é fundamental para facilitar a ação da justiça nos casos judicializados.

Por fim, este estudo permitiu relacionar o conhecimento e as experiências dos especialistas em enfermagem forense, viabilizando a compreensão da trajetória já percorrida, assim como do que ainda precisa ser trilhado com apoio político por meio do desenvolvimento de políticas públicas que incluam o exercício do enfermeiro forense. As competências técnicas e científicas já praticadas pela enfermagem devem ser exercidas com a ampliação do olhar crítico diante de situações de violência, e as perspectivas de avanço para área estão relacionadas à ampliação do conhecimento que viabilizará a ascensão da especialidade. Ainda há dificuldades como o desconhecimento dos profissionais e a falta de envolvimento político para fortalecer a especialidade. Tais dificuldades podem ser justificadas pela dimensão territorial deste país, assim como pelas especificidades culturais de cada região.

# Translation: La enfermería forense brasileña desde la perspectiva de sus especialistas

**DOI:** 10.1590/1980-220X-REEUSP-2024-0402es

**Authors:** Davydson Gouveia Santos, Vanessa Martinhago Borges Fernandes, Morgana Oliveira Citolin, Andreza Goulart Hilleshein, Maria de Fátima Saturnino, Mara Ambrosina de Oliveira Vargas

**Affiliations:** 1 Universidade Federal de Santa Catarina, Programa de Pós-Graduação em Enfermagem, Florianópolis, SC, Brasil.

**Keywords:** Enfermería Forense, Atención de Enfermería, Ciencia y Desarrollo, Rol de la Enfermera, Enfermería Práctica

## Abstract

**Objetivo:**

Comprender la enfermería forense brasileña desde la perspectiva de profesionales especialistas.

**Método:**

Estudio cualitativo, realizado con enfermeras especialistas en enfermería forense. La recolección de datos se realizó mediante un cuestionario electrónico aplicado entre febrero y mayo de 2023, compuesto por preguntas sobre características socioprofesionales y de desempeño. Los datos fueron organizados mediante análisis lexicográfico utilizando IRAMUTEQ, analizados desde la perspectiva del análisis de contenido de Bardin.

**Resultados:**

Participaron 55 especialistas que actuaron en las cinco regiones brasileñas, con mayor participación de las regiones Nordeste y Sudeste, ambas con el 41,8% de los profesionales. La mayoría informó ser mujer, blanca, en su mayoría casada o en una relación estable. A partir de la asociación de las clases presentadas en el dendrograma generado por IRAMUTEQ, se generaron cinco categorías relacionadas con los términos léxicos de la Clasificación Jerárquica Descendente: 1) Creación de la enfermería forense y su desarrollo político en el territorio nacional; 2) Establecimiento de ambientes para el desempeño de la enfermería forense acordes a la realidad nacional/cultural; 3) Competencias ya establecidas en la enfermería forense brasileña; 4) Caminos para el éxito de la enfermería forense brasileña; y 5) Perspectivas de la enfermería forense brasileña.

**Conclusión:**

El estudio permitió contextualizar el escenario actual de la enfermería forense brasileña. Los resultados revelaron la violencia como foco de las acciones desarrolladas por la especialidad. Finalmente, fue posible relacionar los conocimientos y experiencias de los especialistas en enfermería forense, permitiendo comprender el camino ya recorrido y el que aún queda por recorrer, como el desarrollo de políticas públicas que incluyan la práctica de la enfermería forense.

## INTRODUCCIÓN

Las ciencias forenses son un conjunto de bases técnicas y científicas aplicadas a la investigación de crímenes y situaciones de violencia con un enfoque jurídico. El término «ciencias forenses» fue introducido en las ciencias de la salud intencionalmente para formar profesionales en diversas áreas, especialmente en enfermería^(1)^.

En Brasil, la Resolución 389/2011 del Consejo Federal de Enfermería (COFEN) reconoce la enfermería forense como una especialidad de la categoría profesional entre las 65 especialidades existentes^(2)^. La Resolución 556/2017 del COFEN reglamentó el ejercicio de la enfermería forense brasileña, sus áreas de actuación y las competencias técnicas de la especialidad^(3)^.

De acuerdo con esta resolución, las enfermeras forenses pueden trabajar en una amplia gama de situaciones, incluyendo trauma, violencia, abuso sexual y consumo de drogas, así como patologías psiquiátricas. Su trabajo abarca la asistencia a diversos grupos, como agresores, poblaciones vulnerables y el sistema penitenciario. Incluye actividades como el peritaje y la consultoría, además de hacer frente a catástrofes masivas y distintas formas de violencia, a menudo fuera del ámbito hospitalario. Para desempeñar esta función, los enfermeros forenses necesitan tener un título de especialización reconocido por el Ministerio de Educación o por instituciones vinculadas al Sistema COFEN/Consejos Regionales^(3)^.

Entre las competencias fundamentales del enfermero forense se encuentran la conservación de vestigios y de la cadena de custodia, la obtención de datos mediante fotografías y documentación, así como la elaboración de informes y dictámenes para los tribunales. También se encargan de asesorar en casos relacionados con el área forense, incluyendo cuestiones de salud, daños personales, fraudes y otras formas de abuso^(4)^.

El trabajo de las enfermeras forenses es muy diverso y es crucial en la atención a las víctimas de la violencia, ya que suelen ser las primeras en apoyar al paciente en un entorno hospitalario^(4)^. Además, la violencia ha aumentado progresivamente a lo largo de los años, lo que refleja una compleja interacción de factores sociales, económicos, políticos y culturales^(5)^. Como fenómeno sistémico, la violencia influye directamente en los sistemas de salud, seguridad y bienestar, con un impacto negativo en la calidad de vida de la población.

El desarrollo de la enfermería forense en Brasil puede contribuir significativamente a mejorar la calidad de vida de las víctimas y ayudar a prevenir episodios de violencia^(6)^. Las enfermeras desempeñan un papel esencial en la atención a las víctimas de violencia, y son indispensables en la toma de anamnesis, la realización de exámenes físicos y la elaboración de diagnósticos de enfermería. Estas etapas son decisivas para el ingreso en el sistema sanitario^(7)^. Independientemente de su antigüedad en el sector sanitario, las enfermeras reconocen la importancia de la formación forense en el contexto clínico, para garantizar el cumplimiento de los principios éticos y jurídicos y asumir la responsabilidad de reconocer, recoger y conservar las pruebas en el contexto de la atención a usuarios con necesidades físicas, psicológicas y psicosociales complejas^(8, 9)^.

Dado el rol crucial de las enfermeras en la atención y el apoyo a los pacientes, se convierten en una figura central en el proceso de asistencia a las víctimas de la violencia en el contexto de los cuidados. La enfermería forense se distingue por unir el sistema sanitario con el judicial, implicando acciones como búsquedas, investigaciones y cuidados. Además de la práctica clínica, esta área de la enfermería desempeña un papel importante en la obtención de pruebas al participar activamente en la prevención, identificación y lucha contra la violencia, contribuyendo al desarrollo de respuestas a las diferentes situaciones de agresión^(10)^. Aplicando los conocimientos de la enfermería forense, las enfermeras pueden identificar casos de violencia y promover cuidados más humanizados, ya que sus acciones pueden ayudar a romper el ciclo de la violencia. Para ello, deben adaptarse a las necesidades individuales, basarse en la evidencia científica y desarrollarse de acuerdo con las leyes vigentes, las políticas de salud pública y los recursos básicos de enfermería, con el objetivo de reducir el daño causado a las víctimas de la violencia^(11)^.

Teniendo en cuenta lo anterior, y el hecho de que se trata de un área emergente que aún no se ha incorporado a las instituciones sanitarias brasileñas, el objetivo de este artículo fue comprender la enfermería forense brasileña desde la perspectiva de los profesionales especializados. Para ello, se consideraron los aspectos históricos, la realidad actual y las perspectivas de progreso de la especialidad.

## MÉTODO

### Tipo de Estudio

Estudio descriptivo, con abordaje exploratorio y de naturaleza cualitativa. La presentación siguió las recomendaciones del *COnsolidated criteria for REporting Qualitative research* (COREQ)^(12)^. *Localización*

Se utilizó un muestreo de conveniencia y los datos se recogieron mediante la distribución de un cuestionario *en línea* en plataformas virtuales como *correo electrónico, Instagram* ^®^ *, Facebook* ^®^ y grupos de *WhatsApp* ^®^. El cuestionario se puso a disposición en *Google Forms* ^®^, y este enfoque permitió llegar a todas las regiones de Brasil.

### Población y Criterios de Selección

La población del estudio estuvo constituida por enfermeros forenses con experiencia práctica en atención o docencia en Brasil, lo que incluyó personas de diferentes regiones del país y permitió comprender las variaciones regionales.

Los criterios de inclusión para participar en el estudio fueron enfermeros forenses con especialización *lato sensu* en enfermería forense o *pericia* en el área, con experiencia práctica asistencial o docente en Portugal. Los criterios de exclusión incluían profesionales interesados en el área, pero sin cualificación teórica o práctica relacionada con la enfermería forense.

### Definición de la Muestra

Se utilizó la técnica *de bola de nieve*, un muestreo no probabilístico, para reclutar a los participantes. Se utilizaron cadenas de referencias para localizar a posibles participantes. Inicialmente, se invitó a los primeros individuos a través de una referencia de la primera autora de este estudio y, posteriormente, a través de sugerencias de los propios participantes. La recopilación de datos se interrumpió cuando las referencias empezaron a repetirse, sin respuestas adicionales al cuestionario *en línea*. En total, se registraron 63 respuestas y, tras depurar los datos, 55 enfermeras forenses cumplieron los criterios de inclusión y completaron el cuestionario.

### Recolección de Datos

Los datos se recogieron entre febrero y mayo de 2023 mediante un cuestionario estructurado elaborado por los autores, que se dividía en cuatro partes. La primera parte contenía el título del estudio, la invitación a participar, los criterios de inclusión, el formulario de consentimiento informado y las opciones «He leído las condiciones y acepto participar en el estudio» y «He leído las condiciones y no acepto participar en el estudio». Al seleccionar la opción de aceptar, el participante tenía acceso al cuestionario, mientras que si no aceptaba, la invitación se cerraba y *Google Forms* ^®^ le redirigía a una página de agradecimiento. La segunda parte contenía preguntas sobre las características de los participantes. En la tercera parte se aplicó la Técnica de Asociación Libre de Palabras (FWA), que pedía a los participantes que nombraran las cinco palabras que les venían a la mente al pensar en el trabajo de los enfermeros forenses en Brasil. La FWA es una técnica subjetiva utilizada para explorar contenidos latentes no filtrados por la censura. La cuarta parte trató de la aplicación de la enfermería forense en Brasil.

### Análisis y Tratamiento de los Datos

Los datos del perfil socioprofesional se registraron en una hoja de cálculo *de Microsoft*Excel^®^, se verificaron dos veces y se procesaron utilizando el *programa Statistical Package for the Social Sciences* (SPSS), versión 29. El análisis siguió la estadística descriptiva, con medidas de tendencia central (media, mediana, máximo y mínimo) y de dispersión (desviación típica). Los datos obtenidos a través del TALP y sobre el desarrollo de la enfermería forense en Brasil fueron analizados lexicográficamente utilizando el *software Interface de R pour les Analyses Multidimensionnelles de Textes et de Questionnaires* (IRAMUTEQ).

IRAMUTEQ^(13)^ es una herramienta que organiza textos a partir de la similitud entre palabras, permitiendo la creación de clases y categorías que agrupan los términos más significativos. Estos resultados se presentan en forma de nube de palabras y de dendrograma, utilizando la Clasificación Jerárquica Descendente (CJD). Para interpretar los datos de la CJD, se analizaron las formas activas (adjetivos, adverbios, sustantivos, verbos) de cada clase de segmento. Los textos con un valor ≥ 3,26 y un valor p ≤ 0,03433 en la prueba chi-cuadrado (chi^2^) indican significación estadística en la asociación de palabras dentro de su clase.

Tras el procesamiento, se analizó el significado central de los segmentos, destacando las palabras más significativas. Esto permitió categorizar el contenido léxico de las respuestas, organizado en ejes temáticos mediante el análisis de contenido. Este tipo de análisis implica la lectura de las pruebas producidas, la codificación de las unidades de significado, la interpretación y la realización de inferencias, así como el análisis de las categorías temáticas generadas por el IRAMUTEQ, estableciendo conexiones con los marcos conceptuales de la investigación.

Los datos de la cuarta etapa fueron analizados utilizando la técnica de análisis de contenido propuesta por Bardin^(14)^, con el apoyo del *software Qualitative Data Analysis* (QDA) *Miner* ^(15)^. El análisis de contenido comprende el «conjunto de técnicas de análisis de las comunicaciones» e incluye las fases de preanálisis, exploración del material, tratamiento de los datos e interpretación.

En la etapa de preanálisis, los datos se transportaron a QDA *Miner* y se realizaron lecturas flotantes para conocer el texto y seleccionar indicadores para la etapa siguiente. Una vez explorado el material, se codificaron y condensaron las unidades de registro para crear categorías empíricas. A continuación, los datos se procesaron e interpretaron de acuerdo con la lectura sobre el tema.

### Aspectos Éticos

El estudio fue aprobado por el Comité de Ética en Investigación de la Universidad Federal de Santa Catarina, bajo el Informe nº 5.808.287. Todos los participantes firmaron el Formulario de Consentimiento Libre e Informado *en línea*, seleccionando la opción «He leído los términos y acepto participar en la investigación». Se cumplieron todos los aspectos éticos de la investigación con seres humanos. Para garantizar el anonimato, los participantes se identificaron con la abreviatura «EF» (enfermera forense), seguida de un número cardinal en orden ascendente.

## RESULTADOS

Participaron en el estudio 55 (100%) enfermeros, 46 (83,6%) mujeres y nueve (16,4%) hombres, con una edad media de 41 años (DE = 10,5; Mín = 23; Máx = 65). En cuanto al color, 29 (52,7%) se declararon blancas, 18 (32,7%) pardas, siete (12,7%) negras y una (1,8%) amarilla. La mayoría estaban casados o en unión estable (n = 26/47,3%). Además, 22 (40%) eran solteros, seis (10,9%) divorciados y uno (1,8%) viudo. La religión predominante era el catolicismo, citado por 30 (54,5%) participantes; seis (10,9%) dijeron ser protestantes o evangélicos; cinco (9,1%) se declararon agnósticos; cuatro (7,3%) dijeron ser espiritistas; seis (10,92%) profesaban otras creencias cristianas o africanas (cristiano, testigo de Jehová, candomblé, umbanda); y cuatro (7,2%) dijeron ser ateos o no tener creencias religiosas.

En cuanto al tiempo que llevaban como licenciados, 32 (58,2%) declararon tener entre seis y 20 años de experiencia profesional, 18 (32,7%) entre 21 y 40 años, y sólo cinco (9,1%) hasta cinco años. Además, 14 (25,5%) habían realizado un doctorado, diez (18,2%) un máster y 31 (56,3%) una especialización. El perfil formativo de estos profesionales se muestra en la Tabla 1.

**Tabla 1:** Perfil de formación de las enfermeras forenses Florianópolis, SC, Brasil, 2024.

Variables		N	%
**Tiempo de formación en enfermería**	Hasta cinco años de formación	5	9,1
	Seis a diez años de formación	12	21,8
	11 a 20 años de formación	20	36,4
	21 a 30 anos de formación	11	20
	Más de 30 años de formación	7	12,7
	Total	55	100
**Mayor titulación**	Doctorado	14	25,5
	Máster	10	18,2
	Especialista	31	56,3
	Total	55	100
**Año de formación en enfermería forense**	2015	2	3,6
	2016	1	1,8
	2018	9	16,4
	2019	16	29,1
	2020	2	3,6
	2021	8	14,5
	2022	9	16,4
	2023	3	5,5
	2024	5	9,1
	Total	55	100
**Como adquirió el título de especialista en enfermería forense**	Especialización presencial	19	34,5
	Especialización a Distancia (EAD)	11	20
	Prueba de títulos de asociaciones profesionales	20	36,4
	Cursando + cursos libre	5	9,1
	Total	55	100
**Institución que confiere el título de enfermero forense**	ABEFORENSE	8	14,5
	SOBEF	11	20
	IDE – Universidad Cristo Redentor	8	14,5
	Instituto Keynes	6	10,9
	UNIFAJ	5	9,1
	Unyleya	4	7,3
	Otros	13	23,7
	Total	55	100
**Actividad profesional por Región nacional**	Región Centro-Oeste	3	5,5
	Región Nordeste	23	41,8
	Región Norte	1	1,8
	Región Sudeste	23	41,8
	Región Sur	5	9,1
	Total	55	100

Fuente: datos de la investigación. 2023.

Entre los participantes en el estudio, 28 (50,9%) estaban registrados como especialistas en enfermería forense en la organización COREN a la que estaban afiliados. En cuanto a la pertenencia a organizaciones representativas de la especialidad, 20 (36,4%) estaban asociados a la SOBEF, 12 (21,8%) a la ABEFORENSE y 23 (41,8%) no estaban asociados a organismos representativos de la especialidad.

La Técnica de Asociación Libre de Palabras (TALP) se basó en la citación de cinco palabras al pensar en «el trabajo de la enfermería forense en Brasil». Los cognemas evocados fueron registrados en el orden en que fueron mencionados. Un total de 275 palabras/expresiones fueron evocadas por 55 enfermeros, y se identificaron 29 palabras/expresiones distintas con la mayor frecuencia. El *corpus de* evocaciones se analizó en el *programa* IRAMUTEQ mediante la técnica de nube de palabras, que es uno de los métodos de tratamiento de datos aplicados en este estudio. Este método permite presentar gráficamente las palabras más frecuentes, siendo el tamaño de cada término proporcional al número de veces que fue mencionado. Entre las palabras clave más recurrentes durante la recogida de datos, destacó el término «violencia», mencionado por 21 de los participantes. Los términos mencionados menos de dos veces se excluyeron del gráfico. Así se generó el Figura 1.

**Figura 1:** Nube de palabras generada por la Técnica de Asociación Libre de Palabras sobre el papel de la enfermería forense en Brasil. Brasil, 2023.

Fuente: *Software* IRAMUTEQ, 2023.

La nube de palabras se utilizó como punto de partida para analizar los datos. Las palabras menos destacadas indican diferentes aspectos que influyen en el escenario de la enfermería forense, estableciendo conexiones directas con los términos más destacados. Entre los términos más recurrentes se encuentran «violencia» (21), «cuidados» (11), «desafío» (ocho), así como «justicia», «humanización» y «rastro», que se mencionaron siete veces cada uno. El CJD permitió segmentar el *corpus* textual en clases de segmentos textuales (ST) y sus respectivas palabras, destacando las ideas centrales presentes en las respuestas de los participantes. El análisis jerárquico dio como resultado 151 ST, de los que se clasificaron 132, alcanzando una tasa de utilización del 87,42% y formando cinco clases. En otras palabras, se crearon cinco clusters de ST con palabras similares y correlacionadas, pero distintas de las demás.

En el dendrograma, el *corpus* textual se dividió en dos grupos: el primero, formado por las clases II y III, que representan el 18,2% y el 15,2% de los ST, respectivamente; el segundo, formado por una subdivisión de la clase V, que corresponde al 23,5% de los ST, dando lugar a las clases I y IV, que representan el 18,9% y el 24,2% de los ST, respectivamente (Figura 2).

**Figura 2:** Dendrograma de la Clasificación Jerárquica Descendente con palabras significativas sobre el desarrollo de la enfermería forense brasileña. Brasil, 2023.

Fuente: elaboración propia a partir de los datos generados por el *software* IRAMUTEQ, 2023.

Debido al procesamiento del *corpus* con el *software*, se constató que las clases I, IV y V están asociadas entre sí y son léxicamente opuestas a las clases II y III. Se recuperaron los ST de cada *cluster* y se interpretaron en detalle, basándose en los principios del análisis temático, para comprender sus significados centrales y categorizar así las clases. A partir de los resultados presentados en el dendrograma CJD, se describieron las clases categorizadas y se ejemplificaron con extractos del cuestionario.

La clase I (Creación de la enfermería forense y su desarrollo político en el territorio nacional), relativa a los hitos en la creación y desarrollo de la especialidad en Brasil, reunió 25 STs (18,9%) del total del *corpus* analizado, compuestas por palabras y radicales en el rango entre x^2^ = 4,55 (crecimiento) y x^2^ = 36,45 (Brasil), como «creación» (x^2^ = 17,65), «desarrollo» (x^2^ = 13,14) y «especialidad» (x^2^ = 5,84).

Los enfermeros forenses mencionaron el surgimiento y desarrollo de la especialidad, y elementos relacionados de la formación a la práctica profesional para fortalecer el área: [...] *Resolución COFENNº* 556/2017 (EF32); *Elaboración de protocolos y órdenes judiciales* (EF03); [...] creación de proyectos para promover el desarrollo de la especialidad (EF03); [...] creación de proyectos para promover el desarrollo de la especialidad (EF32); [...] creación de proyectos para promover el desarrollo de la especialidad (EF03). [...] *la creación de proyectos para incentivar la creación de cargos de enfermeros forenses en Minas Gerais* (EF20); [...] *ya que con el aumento de los cursos de posgrado, el número de especialistas se hace mayor y el campo se fortalece* (EF50); [...] el profesional de enfermería es un elemento clave en el desarrollo del campo (EF50). [...] *el profesional de enfermería es el primer profesional que se aproxima al paciente en la unidad hospitalaria; por lo tanto, tiene mucho que aportar en la recolección de rastros e informaciones que serán entregadas a la justicia* (EF16); *comencé estudios de posgrado y he quedado encantada con el mundo de la* [enfermería] *forense y con la fuerza de esta especialidad; intento ponerla en todo lo que puedo* (EF17); [...] *estoy segura de que habrá un antes y un después en la sociedad brasileña tras la creación del cargo y el trabajo de las enfermeras forenses en el área* (EF42).

La clase II (Establecimiento de ambientes para el trabajo de enfermería forense de acuerdo con la realidad nacional/cultural) se refiere a los posibles cuidados prestados por la especialidad, a veces caracterizados por la fragilidad de los atendidos. Abarcó 24 STs (18,2%) del total del *corpus* analizado, constituido por palabras y radicales en el rango entre x^2^ = 4,48 (reconocer) y x^2^ = 27,5 (alcance), como «contexto» (x^2^ = 24), «lesión» (x^2^ = 18,56), «identificar» (x^2^ = 13,81) e «intervención» (x^2^ = 6,11).

El análisis reveló la necesidad de que los conocimientos forenses sean aplicados en los ambientes existentes por los profesionales de enfermería, que a veces no aplican los cuidados forenses: [...] *prestar apoyo de consultoría en casos de litigios relacionados con el área forense en el ámbito de la salud, responsabilidad civil por lesiones corporales y otros abusos;* [...] realizar actividades de enfermería *forense* ; [...] realizar actividades de enfermería *forense en el ámbito de la salud, responsabilidad civil por lesiones corporales y otros abusos* . ...] *realizar actividades de enfermería forense penitenciaria con infractores sancionados judicialmente, con enfoque interdisciplinar para la toma de decisiones con el sistema judicial y de seguridad pública, en el ámbito del derecho penal, civil, militar y laboral, con el objetivo de la resocialización;* [... [...] *recoger, cotejar y preservar rastros de víctimas y victimarios en los diferentes contextos de la práctica de la enfermería forense, en los ámbitos prehospitalario, hospitalario y comunitario, en las Unidades Básicas de Salud Penitenciaria* (UBSP) *u otros contextos profesionales, respetando los límites legales aplicables;* [...]*participar en medidas de preservación de cadáveres en el contexto de desastres masivos, disturbios, catástrofes y misiones humanitarias* ...]*recibir a víctimas y víctimas potenciales en situaciones de violencia sexual, llevar a cabo cuidados de enfermería forense y reconocer la importancia de la participación de la víctima en la recogida, recopilación y conservación de restos* [...] (EF18); *Faltan conocimientos de enfermería forense por parte de la población. Es necesario crear puestos de trabajo formales para las enfermeras forenses, así como desarrollar y dar a conocer la enfermería forense como ciencia y actuar como profesional sanitario en los diversos entornos en los que es necesaria* (EF29); y *Llegar a los entornos profesionales* (EF43).

La Clase III (Competencias ya establecidas para la enfermería forense brasileña) presenta los elementos que ya forman parte de las competencias actuales, pero es necesario ir más allá, ampliándolas de acuerdo con las posibilidades que tiene el área. Reunió 20 TSs (15,2%) del total del *corpus* analizado, y se compuso de palabras y radicales en el rango entre x^2^ = 6,34 (recolección) y x^2^ = 35,2 (rastreo), como «información» (x^2^ = 29,1), «protección» (x^2^ = 23,1) y «autor» (x^2^ = 13,98).

Las competencias pueden ampliarse a medida que se extiende el área y aumenta la cualificación de los profesionales que trabajan en los servicios de atención a víctimas y victimarios: *Cuando empecé a estudiar la especialidad* (enfermería forense), *enumeré las competencias de la enfermera forense y añadí algunas: aplicar procesos de enfermería en la atención a la población reclusa en la prevención de malos tratos, violencia sexual y otras formas de violencia; [...] prestar apoyo de consultoría a la población reclusa en la prevención de malos tratos, violencia sexual y otras formas de violencia; [...] prestar apoyo de consultoría a la población reclusa en la prevención de malos tratos, violencia sexual y otras formas de violencia* . ...] *prestar apoyo de consultoría en casos de litigios relacionados con el área forense en el ámbito de la asistencia sanitaria, la responsabilidad civil por lesiones personales y otros abusos; Emitir dictámenes informativos, como consultor forense, sobre la prestación de asistencia sanitaria y los resultados pertinentes;* [. ...] *validar las recogidas de rastros realizadas por los enfermeros forenses* (EF18); [... ] *educar a pacientes y familiares para reconocer las expresiones de violencia y activar la red de apoyo; activar el equipo interprofesional para proteger y abogar por los pacientes evaluados; educación en comunicación no violenta; formar grupos de apoyo y grupos reflexivos en el trabajo basado en género, sexualidad y masculinidad para enfrentar la violencia* (EF5); *Acogida y atención de enfermería a víctimas/perpetradores/familiares; derivación a la red de protección; recogida y conservación de vestigios y exámenes; evaluación y registro de informes periciales; intervenciones y formación a profesionales y población en general para prevenir la violencia* (EF34).

La Clase IV (Caminos para el éxito de la enfermería forense brasileña) reúne acciones que, de ser implementadas, podrían favorecer la consolidación de la especialidad en Brasil. Se compone de 32 ST (24,2%) del total del *corpus* analizado, formado por palabras y radicales en el rango entre x^2^ = 5,79 (SOBEF) y x^2^ = 23,1 (tema), como «reconocimiento» (x^2^ = 21,98), «institucional» (x^2^ = 12,89), «políticas públicas» (x^2^ = 9,59) y «científico» (x^2^ = 9,59).

Los profesionales que realizan actividades prácticas relacionadas con el área forense destacaron su importancia, así como la necesidad de fortalecer las políticas públicas y avanzar en la enseñanza en los diferentes niveles académicos: *Reconocimiento en el ámbito de la investigación/academia* (EF2); *Reconocimiento de la importancia de aplicar los conocimientos forenses en contextos de salud pública* (EF4); *Reconocimiento profesional a través del esclarecimiento de casos*(EF18); *Reconocimiento en relación a otros colegas de las ciencias forenses* (EF30); *Reconocimiento social e institucional; Reconocimiento* social *e* institucional*; políticas públicas; instituciones educativas calificadas para la formación y capacitación; validación de protocolos siguiendo el debido rigor; articulación de servicios y profesionales*(EF34); *Ser reconocido en los organigramas de los servicios de atención y tener mayor protagonismo en el área de violencia* (EF54).

La clase V (Perspectivas de la enfermería forense brasileña) refleja el deseo positivo de evolución de la especialidad, así como las dificultades de los profesionales para consolidarla. Compuesta por 31 ST (23,5%) del total del *corpus* analizado, está formada por palabras y radicales en el rango entre x^2^ = 4,66 (especialización) y x^2^ = 15,93 (necesidad), como «momento» (x^2^ = 15,84), «conocimiento» (x^2^ = 12,71), «trabajo» (x^2^ = 10) y «profesional» (x^2^ = 9,18).

El deseo de que la especialidad crezca se expresó por las dificultades a las que se ha enfrentado el área, pero también por su potencial: *La enfermería forense tiene un enorme potencial de crecimiento y actuación en Brasil debido a la necesidad de acoger y asistir a las víctimas de la violencia tan presentes en nuestro país* (EF29); *Honestamente, son pequeñas. Por lo que dije arriba, infelizmente, Brasil es un país congelado en sus principios y cuesta mucho luchar para conseguir algo, pero me gustaría creer que un día seremos como los EE.UU. y tendremos una profesión de enfermería forense realmente activa* (EF17); *Entrar en contacto con profesionales con conocimiento en el área y expandir la profesión en nuestro estado de Santa Catarina. Actualmente, la enfermería forense se concentra con la mayoría de los profesionales más respetados en el nordeste del país, y necesitamos incluir a las enfermeras forenses en toda la red de salud e instituciones* (EF37); *Muy baja, pues no veo políticas públicas ni autoridades que luchen por nuestra profesión* (EF24); y *Reconocimiento de la especialidad como forma de combatir la violencia y la inclusión de políticas públicas que incluyan a la enfermería forense* (EF47).

## DISCUSIÓN

Este estudio comprendió el proceso de inserción de la enfermería forense en el escenario brasileño, los ambientes en que las prácticas de la especialidad pueden y deben ser ejercidas, así como las posibilidades de ampliación de las competencias establecidas, los caminos que el área necesita recorrer para su consolidación nacional y las perspectivas de los profesionales que se relacionan con la superación de los desafíos existentes para la aplicación del conocimiento.

Los cuidados de enfermería forense implican la aplicación de habilidades prácticas y pensamiento crítico sobre el proceso de enfermería para impactar positivamente en la salud de pacientes afectados por trauma o violencia, a través de la identificación y anotación de evidencias forenses que deben ser presentadas al sistema de justicia penal sobre los cuidados prestados al individuo^(16)^. Las investigaciones sobre las prácticas de enfermería forense se están llevando a cabo en todo el mundo^(16)^. En Brasil, se han realizado estudios sobre las posibilidades de implementación de prácticas de enfermería forense durante los cuidados ya prestados por enfermeros generalistas y especialistas en otras áreas, como urgencias, obstetricia, violencia sexual y sistema penitenciario^(9, 19, 20)^, entre otras. Es necesaria la inclusión de especialistas en los servicios especializados en la atención a personas en situación de vulnerabilidad o violencia, así como a los perpetradores de estas situaciones.

Este estudio coincide con otros en la importancia de dar a conocer la enfermería forense, recientemente regulada en Brasil, así como sus diferentes áreas de actuación^(9, 21)^. Aunque la formación específica en este sector sea aún limitada, los profesionales de enfermería prestan cuidados de acuerdo con directrices establecidas, muchas veces orientados por protocolos institucionales. Se destaca la estrategia utilizada para atender a las víctimas de violencia, y la enfermera desempeña el papel de acoger y orientar a la paciente hacia otras especialidades^(19)^.

La violencia está en el centro del trabajo de las enfermeras forenses, ya sea en la prevención, el control o el tratamiento de las víctimas o de los autores de la violencia. Clásicamente, la violencia es designada por la Organización Mundial de la Salud como un grave problema prevenible de salud pública. Se define como «el uso intencional de la fuerza física o el poder, real o en grado de amenaza, contra uno mismo, contra otra persona o contra un grupo o comunidad, que cause o tenga muchas probabilidades de causar lesiones, muerte, daños psicológicos, trastornos del desarrollo o privaciones»^(21)^. La prestación de cuidados es un reto para los profesionales de la enfermería forense.

La enfermería forense se caracteriza por ser una nueva especialidad que aúna conocimientos científicos y destaca en la investigación de delitos, pero se necesitan conocimientos científicos para desempeñar con éxito esta responsabilidad. Es importante que los profesionales tengan los conocimientos y la sensibilidad adecuados para identificar la violencia y, con su intervención, ayudar a promover la justicia^(22)^. Las enfermeras que se encuentran en la primera línea asistencial prestan los primeros cuidados a las víctimas de la violencia, independientemente del tiempo que lleven trabajando en el sector o de si se consideran capacitadas o preparadas para este tipo de atención. Además, la escasez de recursos humanos y la falta de reconocimiento del papel de la enfermería forense figuran entre los retos de la práctica enfermera^(23)^. La percepción de las enfermeras que recogen, identifican y preservan rastros en el cuidado de pacientes en situaciones de violencia facilita la tramitación legal de los casos que se judicializan.

La enfermería forense es la combinación de la ciencia de la enfermería y los sistemas de salud y justicia. Su trayectoria comenzó en los años 70 en Estados Unidos, cuando un grupo de enfermeras defensoras de los derechos de la mujer luchó por una atención más integral a las víctimas de violación, que incluía la recogida de pruebas médicas. Las enfermeras realizaban exámenes físicos y recogían pruebas forenses cuando atendían a víctimas de violencia sexual, pero no estaban reconocidas como expertas y no se les permitía declarar ante las autoridades judiciales en los tribunales^(23)^.

Con la introducción del programa *Sexual Assault Medical Forensic Exam* en Estados Unidos en la década de 1980, las enfermeras fueron reconocidas como examinadoras, estableciendo un nuevo campo de especialización para la profesión^(24)^. Ante la creciente necesidad de atender a las víctimas de la violencia, fue esencial que las enfermeras desarrollaran nuevas competencias y obtuvieran certificaciones específicas para trabajar en el ámbito forense. Así, empezaron a actuar como examinadores independientes en equipos formados por diversos especialistas, entre ellos policías, fiscales y defensores públicos. Este modelo pionero se originó en California, Estados Unidos, a través de los programas *Sexual Assault Nurse Examiner* y *Sexual Assault Response Teams* ^(25)^.

La formación y el avance de la especialidad de enfermería forense en Brasil representan hitos importantes. Con mayores avances observados en naciones como Portugal, Estados Unidos y Japón, la especialidad se encuentra en una fase incipiente en América Latina y todavía es incipiente en el país. Sin embargo, ya es reconocida oficialmente como una especialización para enfermeros por el COFEN, según lo establecido por la Resolución 389/2011, posteriormente sustituida por la Resolución COFEN 581/2018^(2)^. La enfermería forense en Brasil está regulada por la Resolución 556/2017, que define las directrices para la práctica. Esta resolución establece las áreas de práctica, así como las competencias generales y específicas para las enfermeras forenses^(3)^.

Las enfermeras forenses destacaron los orígenes y el desarrollo de la especialidad, conectando elementos de la formación académica con la práctica profesional para fortalecer esta área. Las ciencias forenses pueden entenderse como un conjunto de especialidades que operan en la interfaz entre lo clínico y lo jurídico, utilizando conocimientos de medicina, biología, ciencias sociales, antropología y criminalística en el ámbito forense.

En enfermería, esta especialización surge del amplio campo de la medicina forense, y crea un enfoque que sitúa a las enfermeras como un socio relevante en el sistema judicial. La enfermería forense abarca el uso de los conocimientos científicos y técnicos de la enfermería en contextos clínicos forenses, uniendo la comprensión jurídica con la atención al paciente. Esta integración constituye un avance significativo en la asistencia a las víctimas de la violencia^(26)^.

La creación de espacios adecuados para la actuación de la enfermería forense tiene en cuenta las particularidades culturales y nacionales y está motivada por el tipo de atención prestada, que a menudo incluye pacientes en condiciones de vulnerabilidad. Este contexto está en consonancia con los estudios que enfatizan el papel de las enfermeras como profesionales a la vanguardia de los cuidados, siendo muchas veces las primeras en recibir a las víctimas de violencia cuando son atendidas en las unidades de salud^(27)^.

A partir de los relatos identificados en este estudio, observamos la importancia de la implementación de los conocimientos forenses en ambientes ya establecidos y por parte de los profesionales de enfermería que, en algunas ocasiones, no utilizan estos conocimientos y no siempre están debidamente preparados para lidiar con estas situaciones, ya que muchos carecen de la formación necesaria para atender a las víctimas de violencia^(4, 26)^. Muchos pacientes que buscan atención médica presentan síntomas clínicos que pueden ser indicativos de violencia. Por lo tanto, la formación de los profesionales de la salud es esencial para identificar posibles casos de violencia a través de la atención prestada a los usuarios.

Por lo tanto, es fundamental promover la formación de los profesionales para que sean capaces de prestar una atención adecuada a las víctimas de violencia que acuden a los servicios sanitarios. Estos profesionales deben estar preparados para ofrecer una atención humanizada y acogedora, tanto a las víctimas como, en algunas situaciones, a los agresores^(27)^. Al desempeñar el papel de educadoras en salud, las enfermeras adoptan una perspectiva única del ser humano, tanto individual como colectivamente^(27)^. Este enfoque les permite prestar cuidados de enfermería orientados a la promoción y la prevención de la salud, que van más allá de la mera ejecución de procedimientos técnicos.

Los profesionales de la salud deben escuchar atentamente, sin prejuicios ni juicios, valorando la autonomía de los pacientes y estableciendo una relación de confianza. La atención integral es esencial para el trabajo de las enfermeras dedicadas a la calidad asistencial, lo que hace absolutamente necesaria una formación cualificada.

Los profesionales que desarrollan actividades prácticas en el área forense destacaron la relevancia y necesidad de su trabajo. También enfatizaron la urgencia de mejorar las políticas públicas y hacer que la enseñanza en este campo evolucione en los diversos niveles académicos, en consonancia con la educación continua en salud, que busca reorientar las prácticas de cuidado. Ese abordaje es fundamental para transformar los procesos de trabajo, a partir de la reflexión crítica de los profesionales sobre la realidad de los servicios y de la búsqueda colectiva de soluciones para los problemas identificados^(27)^. Además, el deseo de desarrollar la especialidad se manifestó en las dificultades mencionadas para consolidarla y ampliar sus posibilidades. Aunque la enfermería forense está ganando terreno en el contexto sanitario mundial, todavía quedan obstáculos que afrontar para organizar los cuidados de forma más eficaz^(27)^.

A lo largo de su formación, las enfermeras forenses desarrollan habilidades y conocimientos específicos que les permiten prestar cuidados en contextos de violencia, y son reconocidas como expertas con especialización en la materia. Su trabajo en el ámbito forense se divide en dos áreas principales: civil y penal. En el ámbito de la medicina forense civil, pueden auditar cuentas médicas, gestionar servicios sanitarios e investigar casos relacionados con la falta de competencia, imprudencia o negligencia en la práctica de la enfermería, incluidos los acontecimientos adversos que pueden producirse durante la atención al usuario^(27)^.

La evolución de la enfermería forense ha mostrado su importante contribución en las esferas penal y civil, destacando la urgente necesidad de su reconocimiento y aplicación en la pericia forense en Brasil. A pesar de que los estudios en este campo aún se encuentran en sus primeras fases, la literatura muestra numerosas oportunidades de actuación, sustentadas en fundamentos científicos y humanísticos, que son cruciales para el esclarecimiento de los crímenes, el apoyo a las víctimas y a los agresores, así como para ofrecer asistencia a las familias. En el contexto civil, el papel de los enfermeros adquiere especial relevancia, lo que les permite realizar auditorías y consultorías especializadas en el ámbito de la enfermería^(28)^.

La investigación sobre el tema, la difusión de conocimientos en el área y la creciente demanda de especializaciones en enfermería forense están directamente relacionadas con el aumento de la violencia en todo el mundo^(29)^. El aumento de las situaciones de violencia ha creado una demanda urgente de formación para los profesionales de enfermería y ha estimulado la búsqueda de una educación preventiva centrada en la violencia interpersonal, así como en la identificación de señales de vulnerabilidad y victimización.

Para fortalecer la posición de los enfermeros forenses en Brasil, es fundamental establecer un diálogo con el poder legislativo con vistas a la creación de normas que reconozcan a los enfermeros forenses como peritos criminólogos. También es esencial divulgar ampliamente la importancia de esta especialidad tanto para la sociedad como para la administración pública. También es necesario incorporar asignaturas específicas en los cursos de pregrado y ampliar los programas de postgrado con contenidos teóricos y prácticos^(28)^.

Las enfermeras forenses pueden desempeñar un papel clave en la medicina forense humanitaria, un área de la ciencia forense centrada en objetivos humanitarios. Esta especialidad puede utilizarse para identificar a víctimas de catástrofes a gran escala, personas desaparecidas como consecuencia de conflictos o víctimas de brotes epidémicos^(30)^. Sin embargo, para que los enfermeros se conviertan en expertos en criminalística en Brasil, es esencial que exista una colaboración con el poder legislativo para crear una legislación que reconozca oficialmente el papel de los enfermeros forenses como expertos en criminalística^(28)^.

Las limitaciones de este estudio incluyen el pequeño número de participantes para un estudio nacional en la mayor categoría sanitaria, que es la enfermería. Sin embargo, demuestra la necesidad de ampliar y dar a conocer esta área de conocimiento. Sus puntos fuertes son la caracterización actual de los profesionales implicados y comprometidos con un área en crecimiento en la enfermería, además de presentar algunas perspectivas tangibles para la especialidad. La implicación de profesionales comprometidos permite establecer estrategias de desarrollo de la especialidad, así como subvencionar futuras acciones para la aplicación de la enfermería forense en los servicios de salud. Otro destaque es el hecho de ser el primer estudio nacional con datos primarios sobre las características de los profesionales de enfermería forense una década después del reconocimiento de la especialidad.

## CONCLUSIÓN

Este estudio permitió contextualizar el escenario actual de la enfermería forense brasileña. Se destaca que la mayoría de los participantes provenían de las regiones nordeste y sudeste del país, lo que evidencia el mayor desarrollo de la especialidad en estas regiones. La especialidad surgió en el Nordeste y su expansión se debió principalmente al apoyo de las entidades representativas de la enfermería forense en Brasil, SOBEF y ABEFORENSE, apoyadas por COFEN.

Los resultados de este estudio revelan que la violencia es el foco de las acciones desarrolladas por la especialidad. La enfermería forense utiliza conocimientos científicos y jurídicos para controlar la violencia y tratar a sus víctimas. Es un reto para los profesionales de los servicios de salud desarrollar cuidados humanizados en medio de tanta violencia e inseguridad profesional. El conocimiento de las prácticas forenses es esencial para facilitar la acción de la justicia en los casos judicializados.

Finalmente, este estudio permitió relacionar los conocimientos y experiencias de especialistas en enfermería forense, posibilitando comprender el camino ya recorrido, así como lo que aún falta por hacer con el apoyo político a través del desarrollo de políticas públicas que incluyan la práctica de la enfermería forense. Las competencias técnicas y científicas ya practicadas por las enfermeras deben ser utilizadas para ampliar su visión crítica de las situaciones de violencia, y las perspectivas de progreso en el área están relacionadas con la ampliación de los conocimientos que permitirán el ascenso de la especialidad. Todavía existen dificultades como la falta de conocimiento entre los profesionales y la falta de implicación política para fortalecer la especialidad. Estas dificultades pueden explicarse por la dimensión territorial del país, así como por las especificidades culturales de cada región.
